# Neural mechanisms of the mood effects on third‐party responses to injustice after unfair experiences

**DOI:** 10.1002/hbm.25874

**Published:** 2022-04-15

**Authors:** Enhui Xie, Mengdie Liu, Jieqiong Liu, Xiaoxue Gao, Xianchun Li

**Affiliations:** ^1^ Shanghai Key Laboratory of Mental Health and Psychological Crisis Intervention, Affiliated Mental Health Center (ECNU), School of Psychology and Cognitive Science East China Normal University Shanghai China; ^2^ Shanghai Key Laboratory of Mental Health and Psychological Crisis Intervention, School of Psychology and Cognitive Science East China Normal University Shanghai China; ^3^ Shanghai Changning Mental Health Center Shanghai China; ^4^ Institute of Wisdom in China East China Normal University Shanghai China

**Keywords:** DLPFC, mood, third‐party response to injustice, unfair experience

## Abstract

Behavioral decision theory argues that humans can adjust their third‐party responses (e.g., punishment and compensation) to injustice by integrating unfair experiences. Typically, the mood plays an important role in such a decision‐making process. However, the underlying neurocognitive bases remain largely unclear. We first employ a modified third‐party justice game in which an allocator split an amount of money between oneself and a receiver. The participants can reapportion the money as observers by choosing from the following three costly options: compensate the receiver, accept the current allocation, or punish the allocator. Then, a second‐party pseudo interaction is conducted where participants receive more (i.e., advantageous unfair experience) or less (i.e., disadvantageous unfair experience) than others. Finally, participants perform the third‐party justice game again after unfair experiences. Here, we use functional near‐infrared spectroscopy (fNIRS) to measure participants' brain activities during third‐party responses to injustice. We find participants compensate more to the receiver after advantageous unfair experience, which involved enhanced positive emotion, weakened sense of unfairness, and is linked with increased activity in the right dorsolateral prefrontal cortex (rDLPFC). In contrast, participants punish more on the allocator after disadvantageous unfair experience, which might primarily stem from their negative emotional responses, strong sense of unfairness, and is associated with significantly decreased activity in the rDLPFC. Our results suggest that third‐party compensation and punishment involved differential psychological and neural bases. Our findings highlight the crucial roles of second‐party unfair experiences and the corresponding mood responses in third‐party responses to unfairness, and unravel the intermediate neural architecture.

## Graphical abstract

Our results suggest that third‐party compensation and punishment to injustice involved differential psychological and neural bases. Our findings highlight the crucial roles of second‐party unfair experiences and the corresponding mood responses in third‐party responses to unfairness, and unravel the intermediate neural architecture.

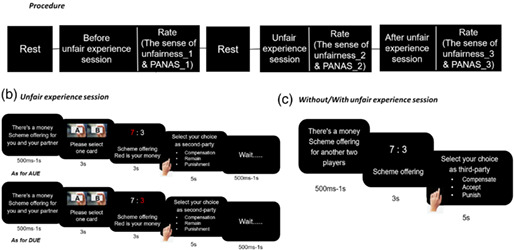

## 1 INTRODUCTION

Imagine a judge who has just experienced unfair treatment (second‐party experience) is dealing with a case involving other's unfair encounters. Then, what decision will the judge make in court of third‐party judicial justice? Would the judge restore justice by compensating the victim who receives unfair treatment or punishing the fairness norm violator? Daily, we may be faced with the situation that our third‐party responses to injustice can be influenced by our personal unfair experiences, but the psychological and neural mechanisms underlying this process remain elusive.

Individuals may be “second‐party” people whose economic payoff are directly affected by the norm violation, while an uninvolved outside party who happens to know that the norm violation occurred is “third‐party” people (Fehr & Fischbacher, 2004). Third‐party responses to injustice may greatly enhance the scope for norms that regulate human behavior (Fehr & Fischbacher, 2004). For example, an allocator may split an unfair amount of money between oneself and a receiver. Then, individuals as unaffected third parties costly punish the allocator or compensate the receiver to injustice (FeldmanHall et al., 2014). Third‐party punishment and compensation are the typical means of ensuring fair and equitable outcomes (Fehr & Fischbacher, 2004; FeldmanHall et al., 2014). Third‐party punishment discourages social norm violation by harming allocators and leaving them worse off (e.g., Deutchman et al., 2020; Karagonlar & Kuhlman, 2013; Sanfey et al., 2003). Third‐party compensation typically addresses the receiver's needs and restores one's reputation, by endowing the victim (the individual who receives less) with the offender's money instead of focusing on punishing the offender (Heffner & FeldmanHall, 2019). Collectively, both third‐party punishment and third‐party compensation are available norm‐enforcing mechanisms.

Although both third‐party punishment and third‐party compensation are the mechanisms of upholding a social norm, these two third‐party responses may be context‐dependent (FeldmanHall et al., 2014). When confronted with an obvious norm violation and given the opportunity to take action either toward the allocator or the receiver, and people typically punish the allocator (Buckholtz et al., 2012; FeldmanHall et al., 2014). Moreover, negative emotion is associated with third‐party punishment such as angry participants punished with highly unfair distributions significantly more than those with a neutral emotion (Gummerum et al., 2016). Recent studies have found preferences for third‐party punishment may be limited, perhaps to contexts where it is not a uniformly preferred method for restoring justice, and third‐party compensation of justice restoration is strongly preferred to punitive measures (FeldmanHall et al., 2014; Heffner & FeldmanHall, 2019). When people are the recipient of an unfair offer and have the option to select from nonpunitive alternatives that satisfy other preferences (e.g., compensation), they prefer to compensate without seeking retribution. Previous evidence has linked more third‐party compensation with individuals in positive moods (Hao et al., 2016). These findings emphasize that, although both third‐party punishment and third‐party compensation are reactions to a social transgression, they may differ in terms of being chosen and depend on the perspective of the deciding agent and mood processing.

Previous studies have demonstrated that the neural mechanisms underlying third‐party punishment and third‐party compensation are similar but different (Stallen et al., 2018). In the response to injustice, brain regions that correlated with third‐party punishment and third‐party compensation are the anterior insula (AIns), anterior cingulate cortex (ACC), dorsolateral prefrontal cortex (DLPFC), precuneus, the bilateral striatum, and ventrolateral prefrontal cortex (VLPFC), and these regions are associated with fairness violations, reward processing and emotional process (Hu et al., 2015; Stallen et al., 2018). Although third‐party punishment and third‐party compensation may be lined with similar regions, different networks are involved in third‐party punishment and third‐party compensation. For example, enhanced ventral striatal activity is associated more strongly with deciding to punish (Guitart‐Masip et al., 2014). More specifically, the right lateral prefrontal cortex (lPFC) during compensation which indicating a cognitive‐control process, instead of revenge‐driven motives, while the left lPFC during punishment is involved in a variety of cognitive and affective processes including integrating emotional information (Hu et al., 2015). These findings provide evidence that both third‐party punishment and third‐party compensation shared a common neuronal basis as third‐party responses to restore justice, and also different networks are involved in the two processes.

As classic psychology and economic theory, behavioral decision theory (BDT) (Simon, 1959) has highlighted the importance of an individual's experience to influence them to be able to change their decision making. In line with this argument, behavioral research has demonstrated that the second‐party events may affect individuals' third‐party responses to violations of fairness norms such as changing their decision to the most punitive option (FeldmanHall et al., 2014; Heffner & FeldmanHall, 2019). Moreover, individuals' responses to injustice are context‐dependent in a “hidden multiplier” game with second‐party asymmetric information in the (they call this moral opportunism) (Van Baar et al., 2019). Evidence from psychology, economics, and neuroscience has demonstrated that people's third‐party responses to injustice are malleable (FeldmanHall et al., 2018; Jones, 1994), but the exact role of second‐party unfair experiences in third‐party responses to injustice remains elusive.

Second‐party unfair experiences are intertwined with our everyday life and even human society which can be divided into when people receive more than others (i.e., advantageous unfair experience) and when people receive less than others (i.e., disadvantageous unfair experience) (Fehr & Schmidt, 1999; Gao et al., 2018; Qiu et al., 2017). After experiencing getting more than others may require not only value representation of the relative economic gain but also advanced social cognition, such as recognizing this norm violation of themselves, and adjusting this violation to bring in long‐term cooperation and benefits (Brosnan & De Waal, 2014; Gao et al., 2018). People with advantageous unfair experiences may generate positive emotions because they benefited themselves economically in the short term (Brosnan & De Waal, 2014). They may be would like to compensate the receiver who receiving less (Dhaliwal et al., 2021). In contrast, the disadvantageous unfair treatment causes significant punishment as a retaliatory behavior to the allocator who received more (Bechtel et al., 2018). Rich economic literature suggests that disadvantageous unfair experiences can easily lead to negative emotions such as anger, and the disadvantageous group may override self‐interest in favor of the punitive option (Fehr & Schmidt, 1999). Therefore, one important outstanding question is whether these two types of unfair experiences lead to different third‐party responses to injustice behaviorally and whether is the role of an important factor on third‐party responses (e.g., the mood) in such a process.

The similarities and differences between second‐party advantageous and disadvantageous unfair experiences have been demonstrated in neural mechanisms (e.g., Bechtel et al., 2018). Neuroimaging results suggest that the advantageous unfair experience is associated with social‐related and mentalizing‐related processes, involving left anterior insula, and dorsomedial prefrontal cortex, but the disadvantageous unfair experience is primarily associated with emotion‐related and conflict‐related processes, involving left posterior insula, right amygdala, and dorsal anterior cingulate cortex (Gao et al., 2018; Yu et al., 2014). Compared with responses to injustice after the advantageous unfair experience, responses to injustice after experiencing disadvantageous unfair treatment may involve more simple processes (Bechtel et al., 2018). Importantly, both types of unfair experience activate the specific prefrontal cortex (PFC). More specifically, the dorsolateral prefrontal cortex (DLPFC) seems to play a critical role in mediating responses to unfair distribution (Güroğlu et al., 2014; Klaus et al., 2012), regions implicate in motivation and morality (Campanha et al., 2011; Hartsough et al., 2020; Knoch et al., 2006). Previous works suggest that the cognitive processing is linked to DLPFC activity, and higher levels of cognitive control make it easy to overcome the strong emotional tendency (Sanfey et al., 2003), whereas lower cognitive demands make it hard to override material self‐interest and emotional reaction to unfair offers (Berna et al., 2010; Klaus et al., 2012). Collectively, previous studies have shown that advantageous and disadvantageous unfair experiences showed similar and differential neural mechanisms respectively. However, it is not clear whether third‐party compensation and third‐party punishment after unfair conditions involve differential neurocognitive mechanisms at the DLPFC.

The present study aims to know about the respective roles of second‐party advantageous and disadvantageous unfair experiences in third‐party responses to injustice, and how these two types of unfair experience influence third‐party responses respectively. Building on the BDT (Simon, 1959), there is much evidence that humans are highly motivated to rebalance the scales of justice after experiencing norm violations (FeldmanHall et al., 2018; Mathew & Boyd, 2011). The present study manipulates the type of unfair experience (Advantageous and disadvantageous) to reveal the effects of unfair experience on third‐party responses to injustice, and we expect that advantageous and disadvantageous unfair experiences would influence third‐party responses to injustice differently. We hypothesize that third‐party compensation and punishment may differ in terms of being chosen and depend on the perspective of the deciding agent and mood processing. Specifically, we expect that people after the advantageous unfair experience would increase the alterations of third‐party compensation, while the disadvantageous group may increase the alterations of third‐party punishment after the unfair experience. Moreover, the neural activation is associated with the third‐party compensation and punishment after unfair experiences (advantageous/disadvantageous) using functional near‐infrared spectroscopy (fNIRS). Given the important role of the DLPFC in the third‐party decision‐making process, it is conceivable that we focused on the DLPFC as the specific area. We expect that third‐party compensation and third‐party punishment after unfair conditions involve differential neurocognitive mechanisms at the DLPFC. To sum up, we aim to explore whether third‐party compensation and third‐party punishment involved differential neurocognitive mechanisms from the perspectives of second‐party unfair experiences, mood processing, and be associated with DLPFC activity.

## 2 MATERIALS AND METHODS

### 2.1 Participants

Using G* Power 3.1 (Faul et al., 2007), based on a small‐to‐medium effect estimated by a meta‐analysis on the effect in the decision making (*f* = 0.19; Balliet et al., 2014) for repeated‐measures ANOVA at an alpha level of .05 with sufficient statistical power of .80 (J. Cohen, 1988), yielded a required sample of 58 at least. In the present study, 60 participants (28 females; mean age 20.1 years; age range: 18–24 years) were recruited. Inclusion criteria were (1) native Chinese speaker, (2) right‐handedness, (3) normal speaking and hearing, (4) no previous or current neurologic/mental disorder according to self‐report. Two same‐sex participants came to the experiment room as a pair. Specifically, all the participants were randomly assigned to experience advantageous unfairness (30 participants in the advantageous group, 14 females; mean age 19.63 years; age range:18–24 years) or experience disadvantageous unfairness (30 participants in the disadvantageous group, 14 females; mean age 20.51 years; age range:18–23 years) in the real two‐player experiment.

All participants provided written informed consent. The study had full ethical approval by the University Committee on Human Research Protection (UCHRP) University Committee on Human Research Protection (HR 351–2019), East China Normal University.

### 2.2 Justice game

**FIGURE 1:**
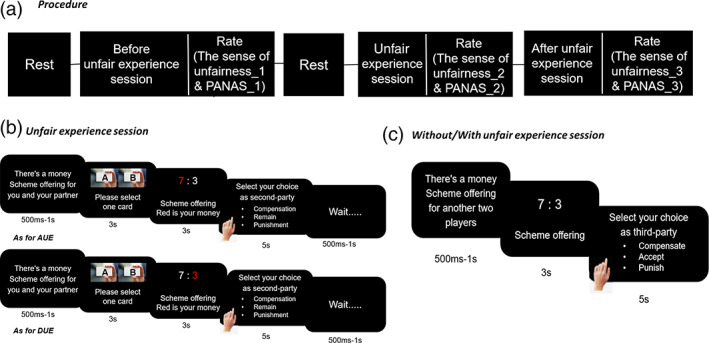
Experimental design. (a) Procedure. The participants took a rest and then finished the justice game under before unfair/unfair/after unfair experience sessions. The sense of unfairness was assessed during different sessions. (b) Unfair experience session. The paired participants were assigned to be advantageous (who receives more in 80% of trials) or disadvantageous (who receives less in 80% of trials) groups at random. The paired advantageous and disadvantageous participants would see the unfair monetary allocation on the screen at the same time (such as “7:3”). In the “advantageous” group, the values were 7 yuan (self, the amounts in red) and 3 yuan (other, the amounts in white; Figure 1b). In the “disadvantageous” group, the value of the gift card was 3 yuan (self, the amounts in red) and 7 yuan (other, the amounts in white). Then, both of the advantageous and disadvantageous participants can select their choice. (c) Before/after unfair experience session. Each participant is labeled as the third party to watch 40 rounds division of 10 yuan. Then, the participants can pay for their fairness preference (for every punish and compensate) or accept the offered scheme (accept). Accept option means agreeing to the proposed split, compensate option means increasing the disadvantageous players' outcome by taking the advantageous players' outcomes, and punish option means decreasing the advantageous players' outcomes instead of increasing the disadvantageous player outcomes, a highly retributive option. The participants have to pay 3 yuan to do all three options. Participants were told that one trial would be randomly selected and the payoff in that trial would be used to pay the participant (i.e., their payoff minus the 3 yuan). PANAS_1, PANAS before unfair experience; PANAS_2, PANAS during unfair experience; PANAS_3, PANAS after unfair experience. The sense of unfairness_1 = the sense of unfairness before unfair experience; the sense of unfairness_2 = the sense of unfairness during unfair experience; the sense of unfairness_3 = the sense of unfairness after unfair experience

The justice game (FeldmanHall et al., 2014, 2018) was adapted to comprise three sessions. In session one—before unfair experience session (Figure 1c)—any division of 10 yuan of other two players was taken on (i.e., 4/6, 3/7, 2/8, 1/9). During this session, participants played a total of 40 trials. The participants can reapportion the money by choosing from the following three options as the third parties; (1) *Accept*: agreeing to the proposed split, the participants have to pay 3 yuan to do this option; (2) *Compensate*: decreasing the advantageous player's outcome (the receiver who received more in the division) while increasing the disadvantageous players' outcomes (the allocator who received less in the division) to match two players' outcomes, the participants as observers have to pay 3 yuan to do this option; or (3) *Punish*: decreasing the advantageous players' outcome while the disadvantageous players' outcome remains unchanged, a highly retributive option, the participants have to pay 3 yuan to do this option. Moreover, participants were told that one trial would be randomly selected and the payoff in that trial would be used to pay the participant (i.e., their payoff minus the 3 yuan).

In session two—unfair experience session (Figure 1b)—any division of 10 yuan of participants was taken on (i.e., 4/6, 3/7, 2/8, 1/9). Participants can determine allocations on their initiative by choosing cards, thus they could be more involved in the study (O. Li et al., 2018). We first raffled two gift cards (Card A and Card B) (Bechtel et al., 2018), and the participants were randomly assigned to be either advantageous or disadvantageous groups by choosing one gift card. The paired advantageous and disadvantageous participants would see the unfair monetary allocation on the screen at the same time (such as “7:3”). In the “advantageous” group, the values were 7 yuan (self, the amounts in red) and 3 yuan (other, the amounts in white; Figure 1b). In the “disadvantageous” group, the value of the gift card was 3 yuan (self, the amounts in red) and 7 yuan (other, the amounts in white; Figure 1b). Both advantageous and disadvantageous participants were respondents, and they were given the option to either compensate or punish or to do nothing. (1) Accept: agreeing to the proposed split; (2) Compensate: decreasing the advantageous player's outcome (the receiver who received more in the division) while increasing the disadvantageous players' outcomes (the allocator who received less in the division) to match two players' outcomes; or (3) Punish: decreasing the advantageous players' outcome while the disadvantageous players' outcome remains unchanged, a highly retributive option. The design here was to create a better advantageous and disadvantageous unfair experience for the participants as second parties, the participants made the choice as the second parties in this session. Moreover, the distribution scheme has been determined in advance, and the choice of cards did not affect the presentation of the scheme. During this session, participants played a total of 40 trials, with 80% trials of advantageous or disadvantageous in randomized order, determining and further fixing the unfair experience group (advantageous or disadvantageous) for the paired participants.

In session three—after unfair experience session (Figure 1c)—any division of 10 yuan of other two players was taken on (i.e., 4/6, 3/7, 8/2, 9/1), and these splits of money were offered by an allocator. During this session, participants played a total of 40 trials. The participants only knew the money information instead of detailed information about the two players such as gender and age. Then, the participants were required to reapportion the money as observers by choosing from the following three costly options: compensate the receiver (who receiving less), accept the current allocation, or punish the allocator (who receiving more).

### 2.3 Procedures

The experiment consisted of resting‐state phases and task phases. In all experimental phases, the neural activity of the participants was recorded with fNIRS.

The resting‐state phases consisted of 2 sessions. In the first 1 min‐resting session (Figure 1a), participants were instructed to relax while keeping their eyes closed without falling asleep and to avoid excessive head motion. For each participant, this initial resting‐state session served as the baseline of before unfair experience session. The second 1 min‐resting session served as the baseline of after unfair experience session.

The task phases included 2 main phases: before unfair experience phase and after unfair experience phase. During the before unfair experience phase, each participant was labeled as the third party to watch 40 rounds division of 10 yuan at the beginning of before unfair experience session (Figure 1c). Then, the participants can decide their third‐party responses to injustice by reducing their payoff (for every *compensate*, *accept*, and *punish*). Finally, the sense of unfairness (1 = extremely fair, 7 = extremely unfair) was assessed by self‐report. Moreover, to assess the role of the mood in unfair experiences influencing third‐party responses, each participant was required to rate the positive and negative emotion scale (PANAS; Watson et al., 1988) on a 5‐point Likert scale before unfair experiences. After the second resting‐state session, after unfair experience phase included 2 main sessions: unfair experience session and after unfair experience session. In the unfair experience session (Figure 1b), the pseudo‐interactive game was carried on with participants as pairs. The paired participants were assigned to be advantageous (who receives more in 80% of trials) or disadvantageous (who receives less in 80% of trials) groups at random. Advantageous and disadvantageous participants were both respondents as the second parties in session two, and they were given the option to either compensate or punish or accept. Then, the participants should report the sense of unfairness (1 = extremely fair, 7 = extremely unfair) during unfair experience session. During the after unfair experience session (Figure 1c), each participant was labeled as the third party to watch 40 rounds division of 10 yuan. Then, the participants can pay for their third‐party responses. Finally, the sense of unfairness (1 = extremely fair, 7 = extremely unfair) and PANAS after unfair experience was assessed.

### 2.4 Behavioral data analysis

The endorsement rate of each third‐party option (*compensate*, *punish*, *accept*) was considered as the index of the third‐party response to fairness violations. Specifically, third‐party responses to injustice were quantified as the ratio of the endorsement numbers of each option to trial numbers (40 for each session) under different sessions. Three mixed‐design repeated‐measures ANOVAs were conducted on each third‐party response to injustice separately, second‐party responses as the covariant variable, type of unfair experience (advantageous/disadvantageous) as a between‐subject variable, and unfair experience session (before/after) as a within‐subject variable. We considered that the general linear model (GLM) analysis may be an approximate analysis because the number of trials was different from subject to subject in the present study. However, Moscatelli et al. (2012) proposed that the data in the GLM analysis should follow the binomial distribution, and the data were independent. We found our dependent variable, the probability of the sum of the three options was equal to 1. The dependent variable was not independent in our study. Our main analyses and results contributed to the understanding of the psychological mechanisms underlying the processing of how unfair experience influenced third‐party responses. The ANOVAs analyses were enough to support our main results to provide more evidence about the difference among the endorsement of three third‐party responses after unfair experience. Thus, we conducted three separate repeated‐measures ANOVAs as our main analyses (FeldmanHall et al., 2018).

A deeper understanding of the participant's third‐party responses in different groups would require factoring in the related variables, such as the mood variable, empathy, and social value orientation. We also conducted a series of analyses on these variables.

### 2.5 fNIRS data acquisition

The brain data acquisition of each participant was simultaneously recorded with an fNIRS recording system using an ETG‐7100 optical topography system (Hitachi Medical Corporation, Japan). The absorption of near‐infrared light (two wavelengths: 695 and 830 nm) was measured with a sampling rate of 10 Hz. The oxyhemoglobin (HbO) and deoxyhemoglobin (HbR) were obtained through the modified Beer–Lambert law. We focused our analyses on the HbO concentration, for which the signal‐to‐noise ratio is better than HbR (Mahmoudzadeh et al., 2013).

One 3 × 5 optode probe set (eight emitters and seven detectors forming 22 measurement points with 3 cm optode separation) was placed over the prefrontal area (http://www.jichi.ac.jp/brainlab/virtual_registration/Result3x5_E.html, see Table S1 for detailed MNI coordinates), which have been previously associated with third‐party fairness preference and decision making in the processing of unfair offers (Berna et al., 2010; Sanfey et al., 2003). The middle optode of the lowest probe row of the patch was placed at Fpz, following the international 10–20 system (Okamoto et al., 2004). The middle probe set columns were placed along the sagittal reference curve. The probe set was examined and adjusted to ensure consistency of the positions across the participants.

### 2.6 fNIRS data analysis

The SPM‐based software package for fNIRS data analyses was based on the GLM (Ye et al., 2009). Its hemodynamic response function (HRF) filter and the wavelet‐MDL (minimum description length) detrending algorithm were used to remove possible noise and physiological interferences (Ye et al., 2009). We generated contrasts (differential effects between Task and Rest phases) per subject using HbO. Then, to separate the brain activities related to the type of third‐party responses to injustice, we extracted the brain activities of third‐party compensation trials and third‐party punishment trials, separately, and computed the averaged brain activities of third‐party compensation trials and third‐party punishment trials for each participant. Finally, we conducted the repeated‐measures ANOVA on each third‐party response to injustice, type of unfair experience (advantageous/disadvantageous) as a between‐subject variable, and unfair experience session (before/after unfair experience) as a within‐subject variable across all channels. The resulting *p* values from all channels were thresholded using a False Discovery Rate (FDR) method that controls the proportion of false positives among the channels that are significantly detected (Benjamini & Hochberg, 1995).

A subsequent Pearson correlation analysis examined the association between the behavioral and neural indices for the related channels. Further, we conducted the linear regression analysis to explore whether the effect of brain activity on third‐party responses to injustice was moderated by the type of unfair experience (advantageous/disadvantageous): third‐party responses to injustice ~ brain activity × the type of unfair experience. We conducted three separate linear regression analyses on each third‐party response to injustice.

### 2.7 Prediction of third‐party responses to injustice

We conducted trial‐by‐trial discriminative analyses to test whether the activation in PFC could discriminate third‐party responses to injustice. First, several lines of research have proposed that brain activation used fNIRS can be used as classification features (Qing et al., 2021), and we extracted the beta values of all third‐party compensation trials. We split high and low compensation groups according to the median value of the compensation rate. A support vector classifier (SVC) based on support vector machines (SVM) was used to classify high and low groups. The LIBSVM toolbox (http://www.csie.ntu.edu.tw/~cjlin/libsvm, Chih‐Chung & Chih‐Jen, 2011) was used to conduct SVC analysis. Secondly, the training data set was trained by a nu‐support vector classifier (nu‐SVC) with the radial basis function (RBF) and nu was set to the default of 0.5. The other parameters were used to adjust the efficiency of the algorithm (Yan et al., 2008). A leave‐one‐out cross‐validation (LOOCV) approach was employed. In this cross‐validation approach, the classifier was trained with *n*‐1 samples and tested on the remaining sample. The procedure was repeated n times. Finally, based on the probability estimates from the SVC model, the area under the receiver operating characteristic curve (AUC) was calculated (Faraggi & Reiser, 2002). Previous work has shown that AUC can effectively quantify the accuracy of the prediction based on synchronized brain activity (S. Cohen et al., 2018). The significance level (threshold at *p* < .05) was calculated by comparing the AUC from the correct labels with 10,000 randomization samples with randomized labels shuffled. The same analysis was conducted for the beta values of all third‐party punishment trials.

### 2.8 Serial mediation model

Serial mediation models were conducted using the Amos 22.0 statistical software to reveal the underlying processes that how unfair experience influences third‐party responses to injustice. The mood and the activation may play crucial roles in the relationship between the sense of unfairness and third‐party responses to injustice after unfair experiences. The PANAS_2 served as the independent variable, the sense of unfairness_2, and the brain activation in task‐related channels as the mediators, and endorsement rate of the third‐party responses to injustice as the dependent variable in the serial mediation models varied in advantageous unfair experience or disadvantageous unfair experience.

## 3 RESULTS

### 3.1 Behavioral results

**FIGURE 2:**
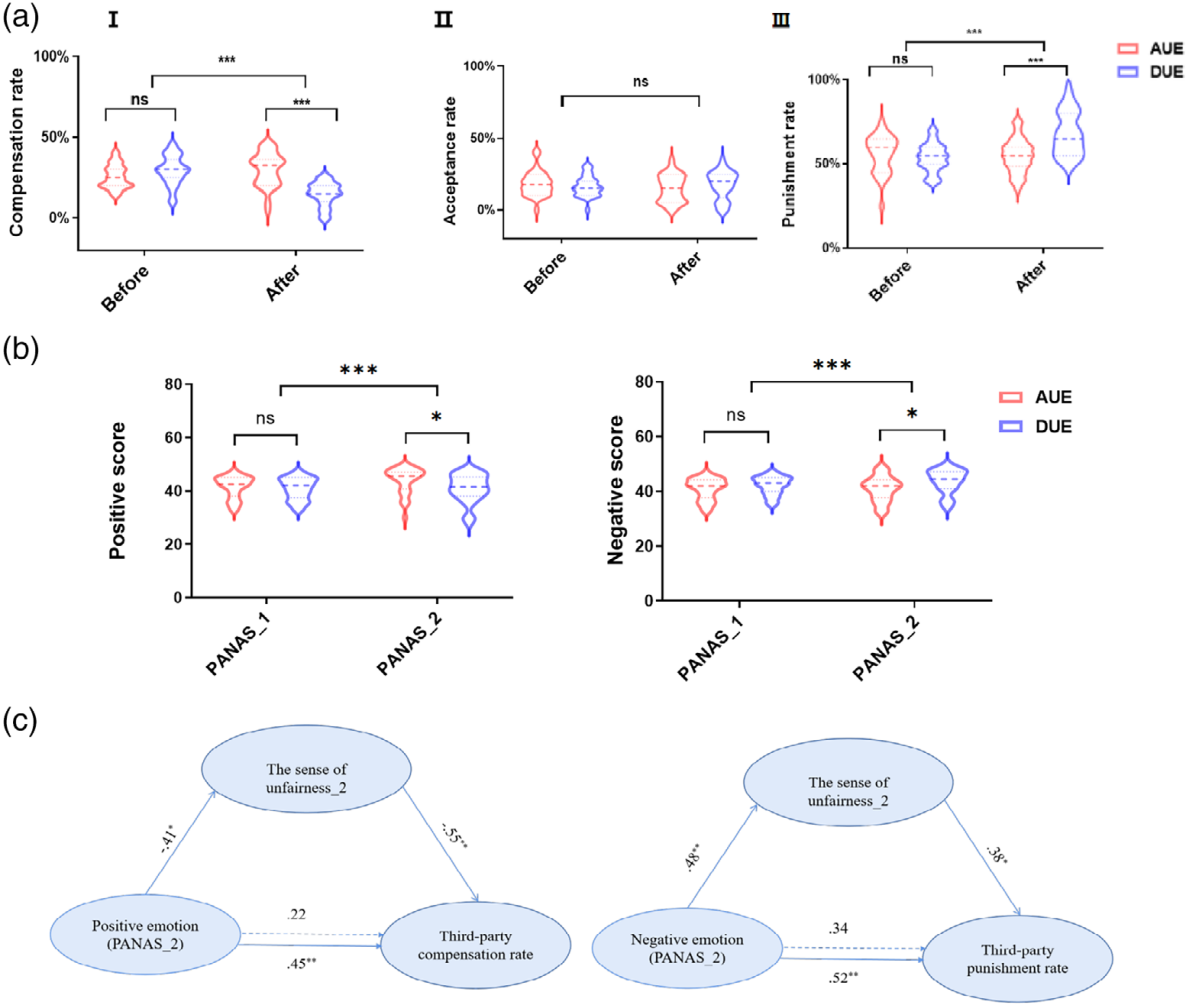
Behavioral results under before/after unfair experiences. (a) Endorsement of third‐party fairness preferences (*compensate/accept/punish* options) in the advantageous and disadvantageous groups under before and after unfair experience sessions. (b) Mood effect between advantageous and disadvantageous unfair before and during unfair experience. (c) Mediation models of the PANAS_2, the sense of unfairness_2, and the third‐party responses. ***p < .001, **p < .01, *p < .05, *ns* is nonsignificant. AUE means advantageous group, DUE means disadvantageous group. Before means before the unfair experience session, after before after the unfair experience session. Error bar in (a) ranges from the min to the max value observed. PANAS_1, PANAS before unfair experience; PANAS_2, PANAS during unfair experience. [Correction added on May 24, 2022, after first online publication: Figure 2 was replaced to fix the values in panel c]

To explore how the type of unfair experience and unfair session influenced third‐party responses, we conducted repeated‐measures ANOVAs on the proportion (%) of trials where the subject made that choice (compensate, accept, punish), with second‐party responses as the covariant variable, unfair experience session (before/after unfair experience) as a within‐subject factor and type of unfair experience (advantageous/disadvantageous) as a between‐subject factor. Importantly, on each trial a subject can only pick *compensate* or *punish* or *accept*, these three choices are mutually exclusive here. The dependent variable was the endorsement rate of third‐party responses. Firstly, we found the second‐party response did not influence our results (*F*s <0.75, *p*s >.390, *η* ^2^_*p*_s <.01). As for the third‐party compensation, the results revealed an interaction between unfair experience session and type of unfair experience (*F*_[1,58]_ = 39.46, *p*<.001, *η* ^2^_*p*_ = .41, Figure 2a, I). Specifically, no significant effect was found in the *compensate* option between advantageous and disadvantageous groups before unfair experience, but the results revealed a significant effect (*F* = 43.81, *p*<.001, *η* ^2^_*p*_ = .43, see Table S2) that advantageous groups prefer *compensate* over the disadvantageous group after the unfair experience. As for the third‐party punishment, the results revealed an interaction between whether experience unfairness and type of unfair experience (*F*_[1,58]_ = 16.31, *p*<.001, *η* ^2^_*p*_ = .22, Figure 2a, III). Specifically, in the *punish* option, no significant effect was found between advantageous and disadvantageous groups before unfair experience, but the results revealed a significant effect (*F* = 27.85, *p*<.001, *η* ^2^_*p*_ = .32, see Table S2) that disadvantageous groups prefer to punish over advantageous groups after the unfair experience. As for the third‐party acceptance, there was no interaction between unfair experience session and type of unfair experience (*F*_[1,58]_ = 2.23, *p* = .147, *η* ^2^_*p*_ = .04, Figure 2a, II).

Moreover, we conducted an independent sample *t* test on the sense of unfairness to ensure the baseline of unfairness between the advantageous group and the disadvantageous group were comparable. We found there was no significant effect between the advantageous group and the disadvantageous group (*t*[58] = 0.17, *p* = .936) on the sense of unfairness_1. To test the difference of the sense of unfairness between the two groups after manipulating the type of unfair experience, an independent sample *t* test was conducted on the sense of unfairness between advantageous and disadvantageous groups. The results showed that there was no significant effect between the advantageous group and the disadvantageous group (*t*[58] = 0.19, *p* = .849) on the sense of unfairness_3. We found experiencing different types of unfair experience both caused high levels of sense of unfairness. Then, a subsequent correlation analysis examined the association between the sense of unfairness under different sessions and the endorsement rate of each third‐party response to injustice. Results revealed that the sense of unfairness_2 showed a significant, negative association with compensation endorsement in the advantageous group (*r*_advantageous (30)_ = −0.53, *p*_advantageous_ < 0.001). Moreover, the sense of unfairness_2 showed a significant, positive association with punishment endorsement in the disadvantageous group (*r*_disadvantageous (30)_ = 0.65, *p*_disadvantageous_ < 0.001). No significant correlation between the sense of unfairness_2 with the acceptance endorsement in either advantageous or disadvantageous group. These findings demonstrated how unfair experience influencing the participants' fairness preferences in the third party varied in advantageous and disadvantageous unfair treatments, in line with the predictions that participants who experienced advantageous unfair treatment preferred to choose to *compensate* option over disadvantageous participants, such preference was linked with the sense of unfairness. However, participants who experienced disadvantageous unfair treatment preferred to choose to *punish* option over advantageous participants, such preference was linked with the sense of unfairness as well.

A deeper understanding of the participant's third‐party responses in different groups would require factoring in the related variables, such as the mood variable, empathy, and social value orientation. To disentangle the impact of the mood variables on the participants' fairness preferences, we first conducted repeated‐measures ANOVAs on the score of positive emotion, with unfair experience session (PANAS_1/PANAS_2) as a within‐subject factor and type of unfair experience (advantageous/disadvantageous) as a between‐subject factor. There was a significant interaction between unfair experience session and type of unfair experience (*F*_[1,58]_ = 8.22, *p* = .006, *η* ^2^_*p*_ = .12, Figure 2b). Specifically, there was a significant difference inpositive emotion between the advantageous and disadvantageous group on the PANAS_1 (*t*[58] = 0.65, *p* = .516) on the PANAS_1. However, our results identified the positive emotion in the advantageous group was significantly higher than the positive emotion score in the disadvantageous groups on the PANAS_2 (*t*[58] = 2.27, *p* = .027). Then, we conducted repeated‐measures ANOVAs on the score of negative emotion, with unfair experience session (PANAS_1/PANAS_2) as a within‐subject factor and type of unfair experience (advantageous/disadvantageous) as a between‐subject factor. There was a significant interaction between unfair experience session and type of unfair experience (*F*_[1,58]_ = 7.36, *p* = .009, *η* ^2^_*p*_ = .11, Figure 2b). Specifically, there was a significant difference of negative emotion between the advantageous and disadvantageous group on the PANAS_1 (*t*[58] = −1.30, *p* = .198) on the PANAS_1. However, our results identified the negative emotion in the disadvantageous group was significantly higher than the negative emotion score in the advantageous groups on the PANAS_2 (*t*[58] = 2.35, *p* = .022). These results provided convictive evidence of mood effect between advantageous and disadvantageous unfair conditions.

Considering individuals may bring in the roles and moods of the second‐party after unfair experience when they made the choice, so we conducted mediation models of the PANAS_2, the sense of unfairness_2, and the third‐party responses. The results suggested a good‐fitted mediation model (CFI = 0.98, TLI = 0.98, RMSEA = 0.03) that positive emotion influenced third‐party compensation rate mediated by the sense of unfairness after advantageous unfair experience (Figure 2c), positive emotion positively predicted third‐party compensation rate (*β* = 0.45, SE = 0.17, *t* = 2.64, *p* = .009), positive emotion negatively predicted the sense of unfairness (*β* = −0.41, SE = 0.17, *t* = −2.40, *p* = .023), the sense of unfairness negatively predicted third‐party compensation rate (*β* = −0.55, SE = 0.16, *t* = −3.48, *p* = .002). Thus, advantageous unfair experience led to positive emotion and less sense of unfairness, thus generating more third‐party compensation which may reduce participants' excess income and use it to make up for the vacancy of their opponents to achieve fairness. Moreover, the results suggested a good‐fitted mediation model (CFI = 0.96, TLI = 0.96, RMSEA = 0.04) that the negative emotion influenced third‐party punishment rate mediated by the sense of unfairness after disadvantageous unfair experience (Figure 2c), negative emotion positively predicted third‐party punishment rate (*β* = 0.52, SE = 0.16, *t* = 3.26, *p* = .002), negative emotion positively predicted the sense of unfairness (*β* = 0.48, SE = 0.17, *t* = 2.92, *p* = .006), and the sense of unfairness positively predicted third‐party punishment rate (*β* = 0.38, SE = 0.18, *t* = 2.14, *p* = .042). Thus, when individuals received less than others, they would perceive more negative emotion and higher sense of unfairness than receiving more than others. Participants in the disadvantageous group tend to choose a spiteful option that may reduce perceived unfairness and vent negative emotions to restore the social norms. In line with the results, participants who experienced disadvantageous unfair treatment preferred to choose to *punish* option.

Additionally, we explored whether empathy, and social value orientation influenced third‐party responses. Previous study has proposed that empathic concern plays an important role in influencing people's choice either to compensate or to punish (Hu et al., 2015). For people who experienced unfair treatment, their empathy was elicited by viewing others' unfair treatment. As a receiver of unfair monetary allocation, people would feel more concerned about people who are suffering from unfair treatment now, and such empathy concern may induce strong emotions which lead people to punish or compensate as the observers. We assessed the empathy using Basic Empathy Scale (BES) (Davis, 1994). We found there was nonsignificant difference in the empathy score between advantageous and disadvantageous groups (*t*[58] = 0.20, *p* = .845). Next, we found the empathy score was positively correlated with the endorsement of compensation after unfair session in the advantageous group (*r* = .48, *p* = .007), and the empathy score was positively correlated with the endorsement of punishment after unfair session in the disadvantageous group (*r* = .37, *p* = .043). Before the experiment, we measured the social value orientation (SVO) of participants using Triple‐Dominance Measure (Murphy & Ackermann, 2014). Participants can be divided into 22 prosocials, 21 individualists and 17 competitors. We found that there was no significant difference between SVO (prosocials/individualists/competitors) × unfair experience session (before/after) based on the endorsement of third‐party compensation (*F* = 2.32, *p* = .107, *η* ^2^_*p*_ = .08), third‐party acceptance (*F* = 0.72, *p* = .492, *η* ^2^_*p*_ = .03), and third‐party punishment (*F* = 2.22, *p* = .118, *η* ^2^_*p*_ = .07). These results did not point to any significant SVO effect.

### 3.2 Brain activation results

**FIGURE 3:**
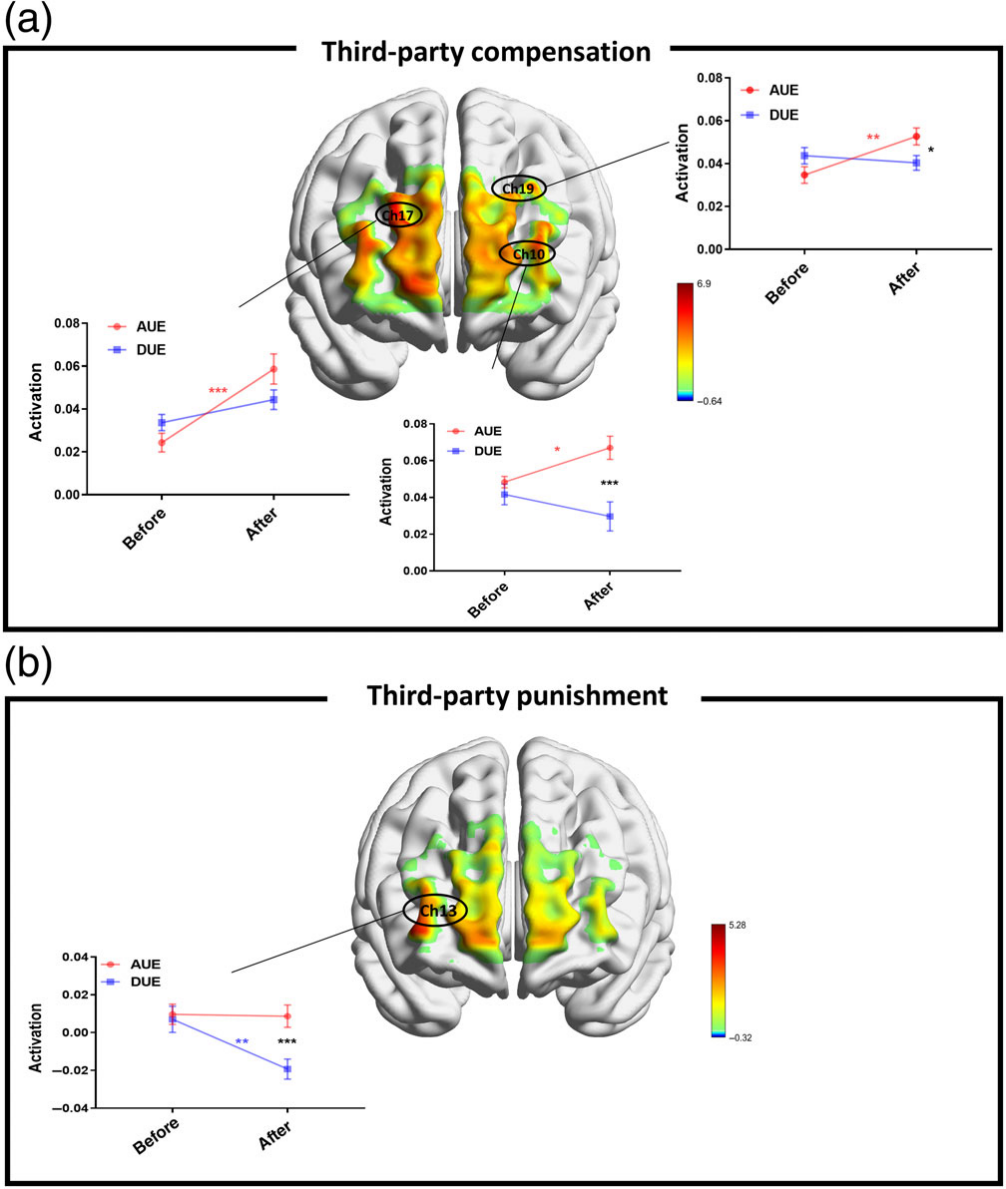
Neural correlates of third‐party compensation and third‐party punishment processing. Central: *F*‐test maps of brain activation generated based on ANOVAs with unfair experience session and type of unfair experience as independent variables. (a) Significant interaction effects were found in CH10, CH17, and CH19 for the third‐party compensation frame. (b) Significant interaction effects were found in CH13 for the third‐party punishment frame. ***p < .001, **p < .01, *p < .05, *ns* is nonsignificant. AUE means advantageous group, DUE means disadvantageous group. Before means before the unfair experience session, after before after the unfair experience session. Error bars reflect 1 SEM

In close correspondence to the behavioral analysis, we established hypotheses for fNIRS analysis showing the potential response patterns for brain regions that were involved in third‐party compensation, third‐party acceptance, and third‐party punishment processing. To understand which brain regions may be involved in processing third‐party responses between advantageous and disadvantageous unfair experiences, we contrasted the fNIRS signal of third‐party punishment/third‐party compensation between conditions before unfair experience and after unfair experience in an ANOVA. Specifically, given the increased third‐party compensation observed after advantageous unfair experience, we hypothesized that brain regions involved in the processing of third‐party compensation would show greater sensitivity to advantageous unfair experience (Figure 3a). We found significant interaction effects at CH10 (*F*_[1,58]_ = 5.41, *p* = .045, FDR corrected, *η* ^2^_*p*_ = .09) in the Orbitofrontal area, CH17 (*F*_[1,58]_ = 6.79, *p* = .038, FDR corrected, *η* ^2^_*p*_ = .11) in the right DLPFC (rDLPFC) area, and CH19 (*F*_[1,58]_ = 6.90, *p* = .036, FDR corrected, *η* ^2^_*p*_ = .11) in the left DLPFC (lDLPFC) area. Further analysis revealed that, as for the advantageous group, higher brain activation was exhibited after experiencing unfairness than before experiencing unfairness in all significant CHs in DLPFC. Given the increased third‐party punishment observed after disadvantageous unfair experience, we hypothesized that brain regions involved in the processing of third‐party punishment would show greater sensitivity to disadvantageous unfair experience (Figure 3b). Significant interaction effect was found at CH13 (*F*_[1,58]_ = 5.28, *p* = .047, FDR corrected, *η* ^2^_*p*_ = .08) in the rDLPFC area. Further analysis revealed that, as for the disadvantageous group, lower brain activation was exhibited after experiencing unfairness than before experiencing unfairness at CH13 in DLPFC. To further ensure CH10, CH17, CH19 were specific to third‐party compensation, we found nonsignificant interaction effects at CH13 (*F*_[1,58]_ = 0.27, *p* = .823, FDR corrected, *η* ^2^_*p*_ = .01) in the rDLPFC area (Figure S1B). To further ensure CH13 was specific to third‐party punishment, we found nonsignificant interaction effects at CH10, CH17, CH19 (*F*s_[1,58]_ < 4.58, *p*s >.145, FDR corrected, *η* ^2^_*p*_ > .07; Figure S1E,G,H).

**FIGURE 4:**
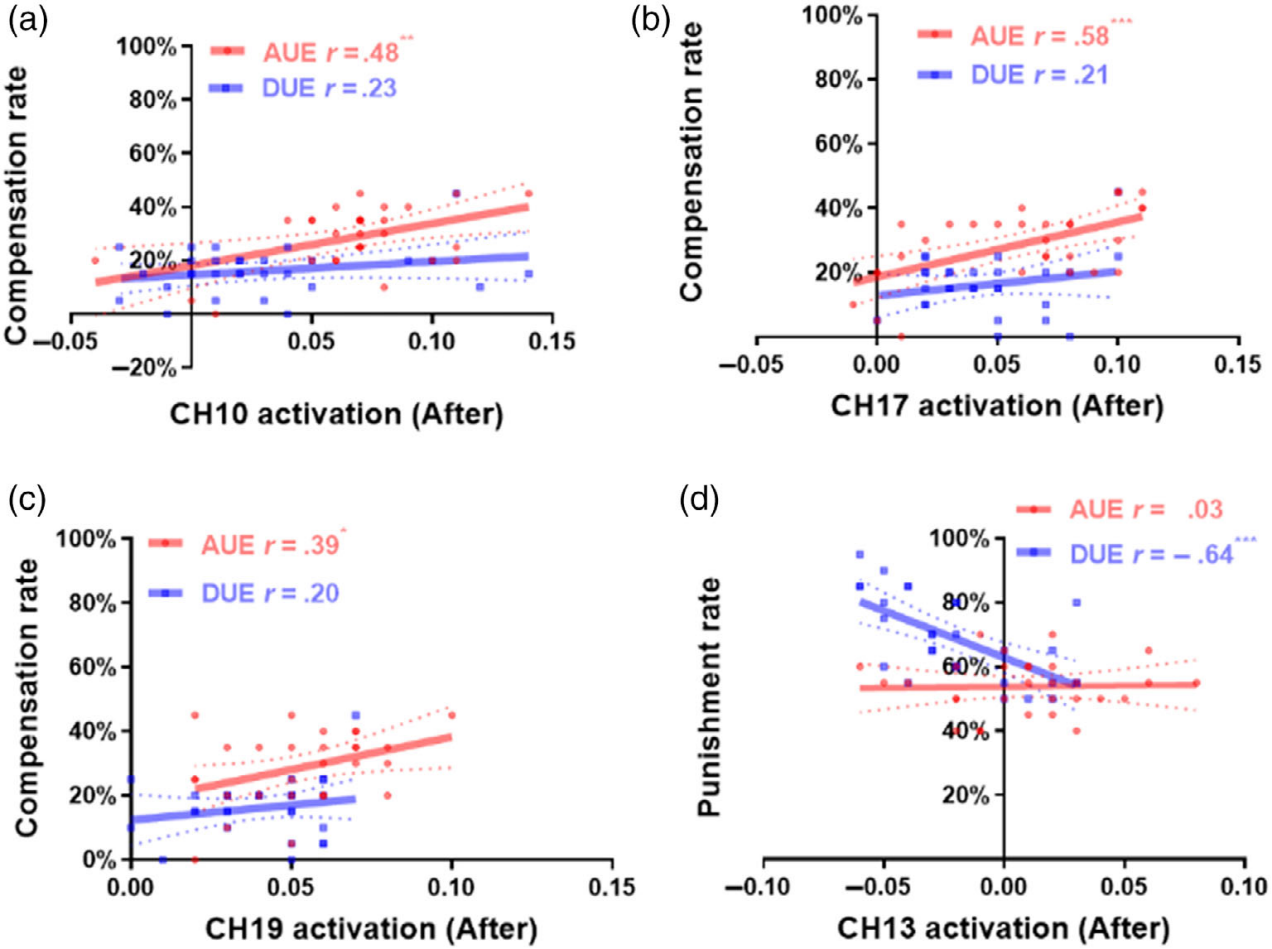
Correlation between behavioral results and corresponding significant CHs. (a–c) Correlation between the endorsement rate of *compensate* and the brain activation at CH10, CH17, and CH19. Only in experiencing advantageous unfairness, the endorsement rate of *compensate was* correlated with the brain activation at CH10. (d) Correlation between the endorsement rate of *punish* and the brain activation at CH13. Only in experiencing disadvantageous unfairness, the endorsement rate of *punish was* correlated with the brain activation at CH13. The dotted line represents the 95% confidence interval. ***p < .001, **p < .01, *p < .05, *ns* is nonsignificant. AUE means advantageous group, DUE means disadvantageous group. Before means before the unfair experience session, after before after the unfair experience session

To further explore the behavioral effect of the fNIRS signal, we linked the fNIRS signal at the individual level during watching offering (before unfair experience session) and the period during which participants choose the preferences (after unfair experience session) to the endorsement rate of corresponding preferences on advantageous and disadvantageous groups. Results revealed that the endorsement rate of *compensate* showed a significant, positive association with the CH10 (*r*_advantageous (30)_ = 0.48, *p*_advantageous_ = 0.009, Figure 4a), CH17 (*r*_advantageous (30)_ = 0.58, *p*_advantageous_ < 0.001, Figure 4b), and CH19 (*r*_advantageous (30)_ = 0.39, *p*_advantageous_ = 0.025, Figure 4c) only in advantageous group under after unfair experience session. No significant association with CH10, CH17, and CH19 in either advantageous or disadvantageous groups was found under before unfair experience session (see details in Figure S2). Furthermore, the endorsement rate of *punish* was negative correlated with the brain activation at CH13 only in the disadvantageous group after unfair experience (*r*_disadvantageous (30)_ = −0.64, *p*_disadvantageous_ < 0.001, Figure 4d), but there was no significant correlation before unfair experience (Figure S2).

Given the correlation between activation and third‐party responses rate, we hypothesized that brain regions involved in the processing of third‐part compensation would show greater sensitivity to the type of unfair experience (advantageous/disadvantageous), which would in turn result in boosted correlation differences between activation and third‐party responses rate in these regions. Based on these hypotheses, we focused on the third‐party responses rate (third‐party compensation rate and third‐party punishment rate) and conducted linear regression analysis to reveal brain regions that were separately involved in the processing of advantageous unfair experience and disadvantageous unfair experience. For the third‐party compensation frame, significant interaction effects were found in the Orbitofrontal area (CH10, *β* = −0.58, SE = 75.61, *t* = −1.95, *p* = .046), rDLPFC area (CH17, *β* = −0.78, SE = 63.07, *t* = −2.17, *p* = .031), indicating the type of unfair experience (advantageous/disadvantageous) could moderate the correlation between activation in these areas and third‐party compensation rate (Figure 4a,b). There was a nonsignificant interaction effect in the lDLPFC area (CH19, *β* = −0.26, SE = 128.30, *t* = −.98, *p* = .269; Figure 4c). For the third‐party punishment frame, significant interaction effect was found in the rDLPFC area (CH13, *β* = −2.05, SE = 2.60, *t* = −4.66, *p* < .001), indicating the type of unfair experience (advantageous/disadvantageous) could moderate the correlation between activation in these areas and third‐party punishment rate (Figure 4d).

### 3.3 Prediction of third‐party responses to injustice

**FIGURE 5:**
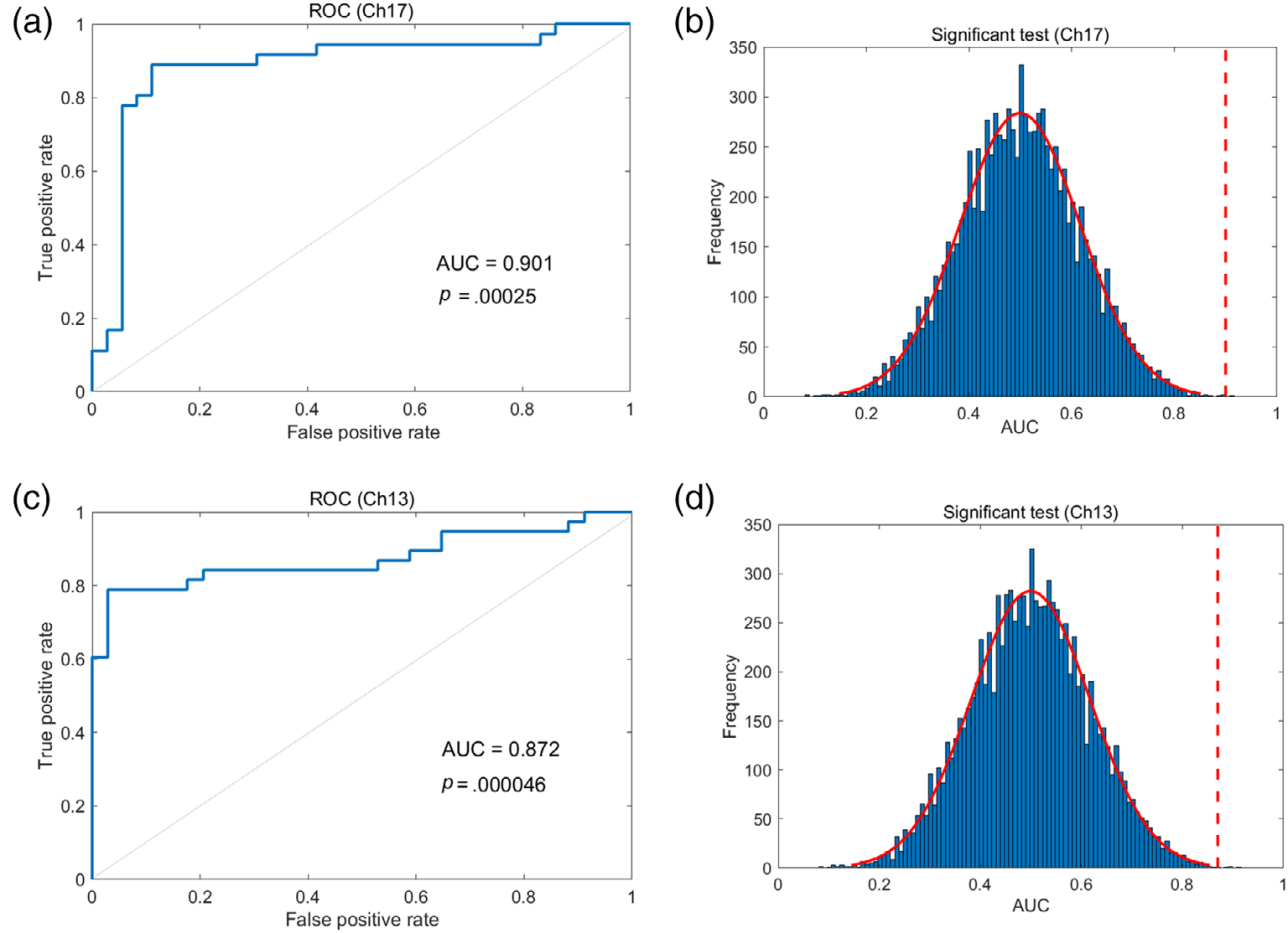
rDLPFC as a specific area can effectively discriminate third‐party responses to injustice. (a) Receiver operating characteristic curve for classification distinguishing high endorsement of the *compensate* from the low endorsement of the *compensate* at CH17. (b) The area under the receiver operating characteristic curve (AUC) is calculated as a metric of classification endorsement of the *compensate* at CH17. (c) Receiver operating characteristic curve for classification distinguishing high endorsement of the *compensate* from the low endorsement of the *punish* at CH13. (d) The area under the receiver operating characteristic curve (AUC) is calculated as a metric of classification endorsement of the *punish* at CH13. The significance level (threshold at *p* < .05) is calculated by comparing the AUC from the correct labels (dotted line) with 10,000 randomization samples with shuffled labels (blue bars)

To further explore our question about how might fNIRS signal during participants after experiencing unfair treatment processing influence third‐party responses to injustice? If activity in neural processing reflects behavioral effect, then the neural activity should discriminate the preference. Moreover, to evaluate if rDLPFC was selectively involved in processing third‐party responses to injustice after unfair experiences, we would expect this region to exhibit good discrimination of third‐party responses after experiencing unfair treatment. We used a trial‐by‐trial SVC from machine learning to discriminate between high and low endorsement rates of third‐party responses to injustice across significant CHs, the beta values at the period during which participants choose the preferences (after unfair experience session). As indicated, the brain activation at CH17 was high and statistically significant for distinguishing high and low third‐party compensation rates after unfair experience (AUC = 0.90, *p* < .001; Figure 5a,b). Moreover, the brain activation at CH10 *(*AUC = 0.69, *p* = .037; Figure S3A,B) and CH19 (AUC = 0.65, *p* = .042; Figure S3D,E) also could distinguish high and low third‐party compensation rate after unfair experience. We extracted the discrimination accuracy at CH10, CH17, and CH19 and conduct a one‐way ANOVA. The results showed a significant main effect (*F*_[2,717]_ = 351.00, *p* < .001, *η* ^2^_*p*_ = .08). Specifically, the multiple comparisons (LSD) result indicated CH17 was statistically better at distinguishing high and low third‐party compensation rates after unfair experience than CH10 and CH19 (*p* < .001). No significant result was found when conducting with CH13 (AUC = 0.45, *p* = .543; Figure S3C). Taken together, the brain activation at rDLPFC (CH17) as the specific neural‐behavioral related index can discriminate the third‐party compensation rate after unfair experience.

The results also showed the brain activation at CH13 was high and statistically significant for distinguishing high and low third‐party punishment rates after unfair experience (AUC = 0.87, *p* < .001; Figure 5c,d). However, no significant results were found when conducting with CH10 (AUC = 0.59, *p* = .284; Figure S3F), CH17 (AUC = 0.36, *p* = .342; Figure S3G), CH19 (AUC = 0.46, *p* = .621; Figure S3H). Taken together, the brain activation at rDLPFC (CH13) as the specific neural‐behavioral related index can discriminate third‐party punishment after unfair experience.

### 3.4 Serial mediation model

We finally turned to our main question about what was the role of the mood in unfair experience influencing third‐party fairness preferences? What was the role of neural activity involve this decision‐making process? Specifically, how might advantageous unfair experience increase individuals' *compensate* options linking with rDLPFC activity? How might disadvantageous unfair experiences increase individuals' *punish* options linking with rDLPFC activity? It was plausible that the sense of unfairness_2 and the activation in the rDLPFC may sequentially mediate the relationship between mood variable (PANAS_2) and third‐party responses to injustice (third‐party compensation or third‐party punishment), we employed a series of serial mediation models to prove this suppose. Moreover, rDLPFC (CH17 and CH13) was regarded as a specific area for *compensate* and *punish* here.

**FIGURE 6:**
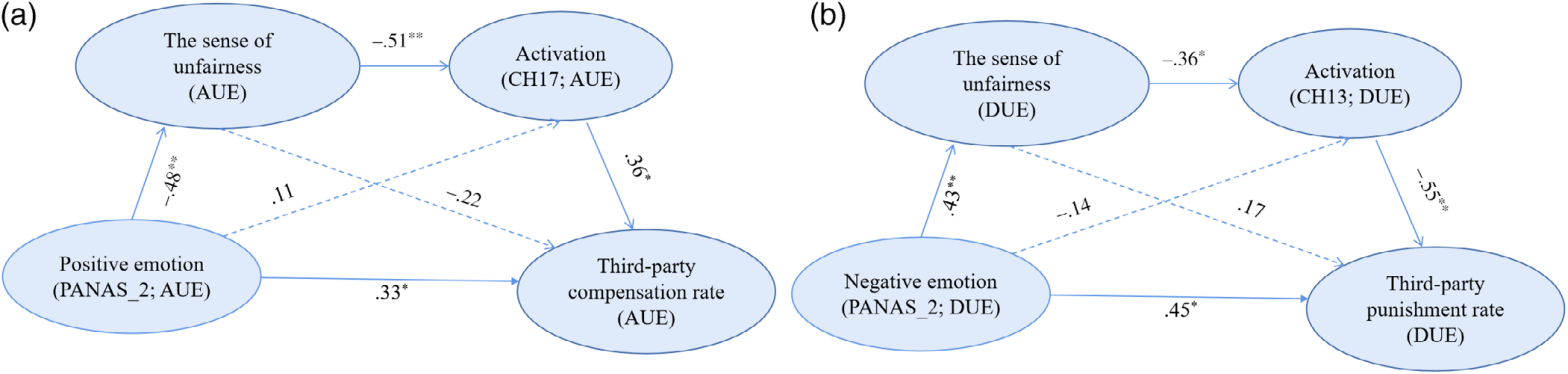
The serial mediation models. (a) A serial mediating effect of the sense of unfairness_2 negatively predicted brain activation at CH17 in the relation of positive emotion (PANAS_2) influencing third‐party compensation rate after advantageous experience session. (b) A serial mediating effect of the sense of unfairness_2 negatively predicted brain activation at CH13 in the relation of negative emotion (PANAS_2) influencing third‐party punishment rate after disadvantageous experience session. *p < .05, **p < .01, ***p < .01

The results revealed a good‐fitted serial mediation model (CFI = 0.97, TLI = 0.94, RMSEA = 0.05) in the advantageous group that positive emotion influenced third‐party compensation rate (Figure 6a). Specifically, positive emotion positively predicted the third‐party compensation rate (*β* = 0.33, SE = 0.14, *t* = 2.22, *p* = .036), positive emotion negatively predicted the sense of unfairness_2 (*β* = −0.48, SE = 0.11, *t* = 3.02, *p* = .007), the sense of unfairness_2 negatively predicted brain activation at CH17 (*β* = −0.51, SE = 0.15, *t* = −3.35, *p* = .001), brain activation at CH17 positively predicted third‐party compensation rate (*β* = 0.36, SE = 0.15, *t* = 2.35, *p* = .024). These findings suggest when receiving more than others, the individuals would increase third‐party compensation after advantageous unfair experience, which may involve enhanced positive emotion and less sense of unfairness because benefitted from the unfair experience, and be associated with increased DLPFC activity. Further, people would choose more third‐party compensation to restore injustice in such mood.

Further, these results indicated a good‐fitted serial mediation model (CFI = 0.94, TLI = 0.95, RMSEA = 0.05) in the disadvantageous group that negative emotion positively predicted brain activation at CH13 in the relation to the sense of unfairness increasing third‐party punishment rate (Figure 6b). Specifically, the results indicated that negative emotion positively predicted third‐party punishment (*β* = 0.45, SE = 0.15, *t* = 2.82, *p* = .001), negative emotion positively predicted the sense of unfairness_2 (*β* = 0.43, SE = 0.15, *t* = 2.80, *p* = .001), the sense of unfairness_2 negatively predicted brain activation at CH13 (*β* = −0.36, SE = 0.15, *t* = −1.97, *p* = .027), brain activation at CH13 negatively predicted the third‐party punishment rate (*β* = −0.55, SE = 0.16, *t* = −3.52, *p* < .001). These results suggest individuals tend to increase third‐party punishment after disadvantageous unfair experience, which may primarily stem from their negative emotional responses and strong sense of unfairness, and such process involving decreased DLPFC activity.

Together, these results revealed a system of mood‐perception‐brain‐behavior reactivity that underlies human third‐party responses to injustice after unfair experiences. Specifically, these results revealed a system in which third‐party compensation was mediated by positive emotion and the sense of unfairness, which was linked with increased rDLPFC activity. These results also revealed a system in which negative emotions after disadvantageous unfair experiences interact to shape neural activity at rDLPFC, which in turn alters third‐party punishment. Thus, these results demonstrated the existence of psychological and neurocognitive mechanisms underlying third‐party compensation and third‐party punishment.

## 4 DISCUSSION

Combining a pseudo‐interactive game that modulated individuals' advantageous and disadvantageous unfair experiences and a variant of just game (FeldmanHall et al., 2018) that enabled us to characterize individuals' changes in third‐party responses to injustice after unfair experience. Here we found that third‐party responses to injustice are shaped by unfair experiences involved us and mood after unfair treatments. More specifically, participants who were exposed to advantageous unfair experience increased their third‐party compensation rate with more positive emotion and those who were exposed to disadvantageous unfair experience increased their third‐party punishment rate with more negative emotion when later deciding how to restore justice as third parties compared to deciding before experiencing unfair treatment. We provided evidence showing that third‐party compensation rate was associated with significantly increased activities in the rDLPFC after advantageous experience, while the third‐party punishment rate was associated with significantly decreased activities in the rDLPFC after the disadvantageous experience. Our results demonstrated these two types of third‐party responses to injustice were associated with differential DLPFC activity. Moreover, brain activation in the rDLPFC was effective in trial‐by‐trial discrimination high and low endorsement rate of third‐party responses to injustice. Individuals tend to increase third‐party compensation after advantageous unfair experience, which may involve enhanced positive emotion and be associated with increased DLPFC activity. In contrast, individuals would alter their third‐party responses into more third‐party punishment after disadvantageous unfair experience, which may vent negative emotion and the sense of unfairness, and involve decreased DLPFC activity.

### 4.1 Distinct roles of the advantageous and disadvantageous unfair experiences in third‐party responses to injustice processing

For people, the theory of BDT and previous studies have demonstrated humans can integrate social contextual information and personal information into decision‐making processes to adjust their responses toward inequity (Giovannelli et al., 2018; Nook et al., 2016; Peysakhovich et al., 2014). Extending findings to the social decision domain, our results suggest that previous unfair experience under the context of (dis)advantageous can alter personal third‐party responses to injustice. Previous studies suggested that third‐party punishment and compensation seem to be driven by a possible motive: people felt empathy with the victim triggering a desire to restore justice (Hu et al., 2015). For people who experienced unfair treatment, their empathy was elicited by viewing others' unfair treatment. People as a receiver of unfair monetary allocation would feel more concerned about people who are in need or suffer from unfair treatment, and such empathy concern may induce strong emotions which lead people to punish or compensate as the observers. We found that people who experienced advantageous unfair cases of monetary allocation preferred third‐party compensation, and such preference was linked with the sense of unfairness. The advantageous people benefited from the previous unfair experiences, and they were more likely to engage in behavior that benefits the group as a whole to be included in future cooperation (Civai et al., 2019; Panchanathan & Boyd, 2004). People who have a weaker sense of unfairness after experiencing advantageous unfair treatment may have a higher ability of cognitive control. They could choose a more rational way to restore justice, that was to compensate the disadvantageous people. People with a weaker sense of unfairness may be prosocial, and they were likely to choose to help the victim in unfair contexts based on the principle of reciprocity (Hu et al., 2015). However, people who experienced disadvantageous unfair cases preferred third‐party punishment, and such preference was linked with the sense of unfairness. They were victims with strong sense of unfairness and higher negative emotion from the previous unfair experience, and third‐party punishment alleviated negative emotions triggered by being treated unfairly (Pillutla & Murnighan, 1996).

### 4.2 The mood effects of unfair experiences on third‐party responses to injustice

Although third‐party responses have been extensively studied in laboratory settings (Chavez & Bicchieri, 2013; Fehr & Fischbacher, 2004), our article contributes to the literature by comparing third‐party compensation and third‐party punishment and underlying their neural activities. Decades of research have examined third‐party responses to injustice under third‐party context instead of combining them with second‐party experience or event (Fehr & Fischbacher, 2004), the current demonstrated second‐party unfair experiences play important roles in the third‐party decision‐making process and such process was related with the mood. Our mediational findings explained how the mood effects of unfair experience on third‐party responses to injustice occurred. This explanatory path suggests that economic interaction will distinguish different roles (advantageous side and disadvantageous side), and such roles would be brought into the perspective of a third party and thus alter people's third‐party responses to injustice. Consistent with previous work (Bechtel et al., 2018), receiving more benefits than others shapes people into a profiteer in an unfair environment. When people perceived positive emotions and less sense of unfairness, which shall be manifested as increased activity in the rDLPFC on the neural level and further led to reducing their interests to compensate the people who receiving less. On the other hand, the disadvantageous unfair experience made people feel like a victim instead of a pure observer in the third party which aligned with previous literature (Fehr & Gachter, 2002; FeldmanHall et al., 2018). Such disadvantageous unfair experience may generate negative emotion and a sense of unfairness which further manifested as decreased activity in the rDLPFC on the neural level and generates more third‐party punishment.

### 4.3 The mood modulates neural activity after unfair experiences

Our findings revealed a mechanism by which unfair experience influenced third‐party responses occurred: the mood (e.g., positive emotion and negative emotion). Based on the theory of decision making, emotion played an important role in decision making. Previous studies provided evidence of these decision‐making theories, for example, Gummerum et al. (2016) found that negative emotion was associated with third‐party punishment such as angry participants punished highly unfair distributions significantly more than those in a neutral emotion. Moreover, previous evidence has linked more third‐party compensation with individuals in positive moods (Hao et al., 2016). Previous theories and studies have linked unfair experiences with emotional processing (Civai et al., 2010; Fehr & Schmidt, 1999). We found previous unfair paradigm studies in which the participant was a passive receiver (Chang & Sanfey, 2013). However, the participant was a participative receiver in the current study. The mood effects were purely caused by unfair experiences. Our results showed people after advantageous unfair experiences would feel more positive emotion than before advantageous unfair experiences because they benefited from the unfair situation. Meanwhile, people after disadvantageous unfair experiences would feel more negative emotions than before disadvantageous unfair experiences. Although previous research studied third‐party responses with the mood variable (Gummerum et al., 2016; Hao et al., 2016), none revealed underlying neural activity. Our results allowed us to disentangle the separate contributions of the elements of the positive emotion and negative emotion in influencing the rDLPFC activity. We found positive emotion after advantageous unfair experience was correlated with rDLPFC activity and negative emotion after advantageous unfair experience was involved in the rDLPFC activity. Our findings provided evidence for the involvement of the rDLPFC in emotional processing. Here, we provided neural evidence that DLPFC, a region implicated in emotional processing, cognitive control, social norm compliance (Gao et al., 2018), contributed to the reaction to the integrated context‐dependent information modulating by emotional processing.

Given the potential relationship between emotion and unfair contexts (Hao et al., 2016), it was possible that unfair experiences induced the sense of unfairness, and then emotion, then the brain activity, and finally behavior. We also ran the mediation models that the PANAS_2 was taken as a mediator, and the sense of unfairness_2 as the independent variable. The results suggested bad‐fitted mediation models that the sense of unfairness influenced third‐party responses mediated by emotion after unfair experience (Figure S4). These SEM results showed that the possible hypothesis could not explain the data well in the present study. Therefore, we were unable to build the pathway that unfair experiences induced the sense of unfairness, and then emotion, then the brain activity, and finally behavior. Future studies are advocated specifically designed to evaluate such possibilities by detecting more emotion‐related regions such as ACC.

### 4.4 Third‐party responses to injustice are associated with distinct brain activity

Neuroimaging results further show that there is a common neural response in the DLPFC to third‐party responses to injustice with unfair experiences. In reference to previous neuroimaging researches on decision making, our findings align with the early works that DLPFC, a region implicated in cognitive control and social norm compliance, contributes to the adjustment of responses to injustice in social contexts (Miller & Cohen, 2001; Sanfey et al., 2003). Previous studies employed a range of approaches, such as tDCS, TMS, and functional connectivity analysis, revealing the activity of the DLPFC: the increased activation was involved in decision making, reward processing, risk‐taking, and social cognition processing, whereas the decreased activation was involved in lower cognitive processing which may lead to emotional reactions, especially negative reactions (Georgii et al., 2017; Tassy et al., 2012). Extending this engaging function of DLPFC, our results demonstrate that the processing of third‐party responses to injustice exhibits a differential activity within the rDLPFC, such that increased activity was predominantly involved in third‐party compensation and decreased activity was predominantly involved in third‐party punishment. Given the increased DLPFC activity was linked with value economic gain and emotion, prosocial and reciprocity, and long‐term cooperation (Brosnan & De Waal, 2014; Ferreira et al., 2012; McAuliffe et al., 2017), our results showed third‐party compensation that may place higher cognitive and emotional demands on evaluative processing (Klaus et al., 2012) was linked with increased brain activation in the DLPFC. Decreased brain activation in the rDLPFC has previously been shown to be linked to lower cognitive processing which may lead to failure to resist the emotional responses to unfairness. In reference to previous neuroimaging research on moral decision making, our findings align with the early work, decreased brain activation involved in third‐party punishment.

To sum up, our study thus contributes to demonstrating the mood effect on the role of rDLPFC in third‐party responses to injustice after unfair experience that pointing more third‐party compensation to advantageous experiences with more positive emotion and less sense of unfairness, such cognitive process was linked with increased rDLPFC activity. However, people's third‐party punishment for the disadvantageous experience was driven by negative emotion linked with decreased rDLPFC activity.

The results of the discriminant analyses showed the power of the rDLPFC in discriminating third‐party responses to injustice using SVM. In line with previous research (Miller & Cohen, 2001), we found the rDLPFC was the specific region linked to the third‐party responses to injustice processing. The brain activation in the rDLPFC was able to distinguish the level of the third‐party responses to injustice (third‐party compensation and third‐party punishment) on a trial‐by‐trial basis using SVC. Moreover, a growing number of studies have used the combination of machine learning and brain activity measurement in social neuroscience. These findings were revealing that brain activity served as a reliable neural classification feature.

### 4.5 Implications and future directions

Although brain activity in the most important region (PFC) can be detected here, spatial resolution and brain depth are restricted in fNIRS, limiting measurements to the cortical surface (Huppert et al., 2017). Structures such as emotional systems and the reward systems, which reputedly play a role in decision making (Gao et al., 2018; Sanfey et al., 2003), are thus not detectable. Our results suggest that the strength of neural activities in DLPFC may be modulated by the moods and different emotions (e.g., positive emotion and negative emotion) led to different paths of influencing DLPFC activity. Future research is needed to conduct concurrent fMRI‐fNIRS to detect what is emotional processing in neural adjustment to DLPFC can be applied to other social contexts that influence third‐party responses and social norms. Second, given that we used the between‐subject design of unfair conditions in the current study. There are not enough numbers for each participant to explore the dynamic process yielding from third‐party compensation to third‐party punishment influenced by unfair conditions and mood effects. In the future, it is worth concerning about dynamic process yielding from third‐party compensation to third‐party punishment influenced by unfair condition and mood effect. Finally, in the current study, we set the compensate option based on the previous literature (Heffner & FeldmanHall, 2019). Although both third‐party compensation and punishment are reactions to a social transgression, these strategies differ substantially in terms of output: punishment discourages social norm violation by harming offenders and leaving them worse off, whereas compensation focuses on victims instead of only focusing on punishing the offenders, signaling altruism and prosociality (Heffner & FeldmanHall, 2019). Thus, we attempted to set compensate option as increasing the disadvantageous players' outcome by taking the advantageous players' outcomes in our experiment. We told the participants the difference between compensation and punishment: punish option meant decreasing the disadvantageous players' outcome, whereas compensate option meant decreasing the disadvantageous players' outcomes, and increasing the advantageous players' outcome at the same time. However, it is hard to separate the compensation and punishment if they coexisted when choosing Compensate choice. Therefore, further studies are needed to pay more attention to setting the options.

In summary, our findings shed light on the mood effect of unfair experiences are taken into account in third‐party responses to injustice and provide psychological and neurophysiological evidence of processes for the alteration of third‐party responses to injustice involved in the contexts with advantageous/disadvantageous unfair experiences. Our findings suggest that unfair experiences enhanced third‐party compensation or third‐party punishment. Moreover, our results suggest that these two types of third‐party responses to injustice may be associated with differential dorsolateral prefrontal cortex (DLPFC) activity. Moods (e.g., positive emotion and negative emotion) affected both the sense of unfairness and the underlying brain activity: Individuals tend to increase third‐party punishment after disadvantageous unfair experience, which may primarily stem from their negative emotional responses, strong sense of unfairness, and involve decreased DLPFC activity. In contrast, individuals tend to increase third‐party compensation after advantageous unfair experience, which may involve positive emotion, the weaker sense of unfairness, and be associated with increased DLPFC activity. Our behavioral and brain imaging findings suggest that the mood provides a cognitive basis for the mood‐perception‐brain‐behavior reactivity that underlies human third‐party responses to injustice.

## CONFLICT OF INTEREST

The authors declared that they have no conflict of interest.

## AUTHOR CONTRIBUTIONS

**Enhui Xie**: Conceptualization, Formal Analysis, Investigation, Writing—Original Draft Preparation, Writing—Review & Editing. **Mengdie Liu**: Conceptualization, Investigation. **Jieqiong Liu**: Writing—Review & Editing. **Xiaoxue Gao**: Investigation, Supervision, Writing—Original Draft Preparation, Writing—Review & Editing. **Xianchun Li**: Conceptualization, Funding Acquisition, Supervision, Writing—Review & Editing.

## Supporting information


**Table S1** MNI coordinate Position of 3 × 5 optode probe set
**Table S2**. Post hoc test of each third‐party response to injustice.

**Figure S1** Significant channels' activation. ***p < .001, **p < .01, *p < .05, *ns* is nonsignificant. AUE means advantageous group, DUE means disadvantageous group. Before means before the unfair experience session, After before after the unfair experience session. Error bars reflect 1 SEM.

**Figure S2** The endorsement rate of third‐party compensation was nonsignificant association with CH10 (*r*_advantageous (30)_ = 0.32, *p*_advantageous_ = 0.085; *r*_disadvantageous (30)_ = 0.35, *p*_disadvantageous_ = 0.069; Figure S3A), CH17 (*r*_advantageous (30)_ = 0.29, *p*_advantageous_ = 0.117; *r*_disadvantageous (30)_ = 0.30, *p*_disadvantageous_ = 0.095; Figure S3B), and CH19 (*r*_advantageous (30)_ = 0.20, *p*_advantageous_ = 0.281; *r*_disadvantageous (30)_ = 0.20, *p*_disadvantageous_ = 0.274; Figure S3D) in either advantageous or disadvantageous groups under before unfair experience session. The endorsement rate of third‐party punishment was nonsignificant correlated with the brain activation at CH13 (*r*_advantageous (30)_ = 0.19, *p*_advantageous_ = 0.313; *r*_disadvantageous (30)_ = −0.28, *p*_disadvantageous_ = 0.131; Figure S3C) in either advantageous or disadvantageous groups under before unfair experience session. Before means before advantageous unfair experience or disadvantageous unfair experience.

**Figure S3** Discrimination of third‐party fairness preferences by brain activation.

**Figure S4** The mediation models that the PANAS_2 was taken as a mediator, and the sense of unfairness_2 as independent variable. (A) The results suggested a bad‐fitted mediation model (CFI = 0.32, TLI = 0.45, RMSEA = 0.31) that the sense of unfairness influenced third‐party compensation rate mediated by positive emotion after advantageous unfair experience. (B) The results suggested a bad‐fitted mediation model (CFI = 0.36, TLI = 0.43, RMSEA = 0.30) that the sense of unfairness influenced third‐party punishment rate mediated by negative emotion after disadvantageous unfair experience.

## Data Availability

The data that support the findings of this study are available from the corresponding author upon reasonable request.
