# Supplementary material for: Neural mechanisms of the mood effects on third‐party responses to injustice after unfair experiences
Source: Hum Brain Mapp. 2022 Apr 15;43(12):3646–61. doi: 10.1002/hbm.25874 (PMC9294295; doi:10.1002/hbm.25874)
Supplement: Supplementary file 1 — Table S1 MNI coordinate Position of 3 × 5 optode probe set Table S2. Post hoc test of each third‐party response to injustice. [file HBM-43-3646-s005.docx]

**Table S1.** MNI coordinate Position of 3 ​× ​5 optode probe set

|  | **MNI coordinate Position** | | |  |  |
| --- | --- | --- | --- | --- | --- |
|  | **x** | **y** | **z** | BrodmanArea(Chris rorden' MRIcro) | **Percentage** |
| **CH01** | -35.863 | 62.609 | -7.256 | 10 - Frontopolar area | 0.586 |
| **CH02** | -12.874 | 71.461 | -3.231 | 11 - Orbitofrontal area | 0.771 |
| **CH03** | 15.016 | 71.070 | -3.320 | 11 - Orbitofrontal area | 0.615 |
| **CH04** | 38.223 | 63.018 | -7.027 | 10 - Frontopolar area | 0.622 |
| **CH05** | -45.367 | 52.591 | 1.121 | 46 - Dorsolateral prefrontal cortex | 1.000 |
| **CH06** | -24.366 | 67.884 | 8.478 | 10 - Frontopolar area | 0.973 |
| **CH07** | 2.131 | 67.978 | 9.096 | 10 - Frontopolar area | 1.000 |
| **CH08** | 26.878 | 67.978 | 8.452 | 10 - Frontopolar area | 0.934 |
| **CH09** | 46.726 | 53.149 | 1.723 | 46 - Dorsolateral prefrontal cortex | 1.000 |
| **CH10** | -35.323 | 57.945 | 18.709 | 46 - Dorsolateral prefrontal cortex | 0.917 |
| **CH11** | -12.725 | 66.800 | 21.616 | 10 - Frontopolar area | 1.000 |
| **CH12** | 14.488 | 67.954 | 22.793 | 10 - Frontopolar area | 1.000 |
| **CH13** | 37.658 | 58.539 | 18.499 | 46 - Dorsolateral prefrontal cortex | 0.789 |
| **CH14** | -45.008 | 41.702 | 27.391 | 45 - pars triangularis Broca's area | 0.922 |
| **CH15** | -23.406 | 56.207 | 32.665 | 46 - Dorsolateral prefrontal cortex | 0.655 |
| **CH16** | 1.510 | 59.220 | 34.097 | 10 - Frontopolar area | 0.533 |
| **CH17** | 25.747 | 57.016 | 32.917 | 46 - Dorsolateral prefrontal cortex | 0.505 |
| **CH18** | 47.084 | 41.584 | 27.844 | 45 - pars triangularis Broca's area | 0.944 |
| **CH19** | -34.525 | 40.245 | 41.748 | 9 - Dorsolateral prefrontal cortex | 0.913 |
| **CH20** | -11.211 | 50.382 | 45.417 | 9 - Dorsolateral prefrontal cortex | 1.000 |
| **CH21** | 13.133 | 50.434 | 45.722 | 9 - Dorsolateral prefrontal cortex | 1.000 |
| **CH22** | 36.155 | 39.804 | 42.142 | 9 - Dorsolateral prefrontal cortex | 0.968 |

**Table S2.** Post-hoc test of each third-party response to injustice.

| **Third-party response** | **Group** | Endorsement rate (*M±SD*) |
| --- | --- | --- |
| Third-party  compensation | Before AUE | 25.67±7.28 |
|  | After AUE | 30.17±10.38 |
|  | Before DUE | 29.50±9.22 |
|  | After DUE | 14.67±7.54 |
| Third-party  acceptance | Before AUE | 18.83±8.68 |
|  | After AUE | 15.33±9.28 |
|  | Before DUE | 16.17±7.03 |
|  | After DUE | 17.00±9.97 |
| Third-party  punishment | Before AUE | 55.50±11.10 |
|  | After AUE | 53.50±10.13 |
|  | Before DUE | 54.33±8.38 |
|  | After DUE | 68.33±12.91 |
| The delta value of  third-party response  (After-Before) | Third-party  compensation (AUE) | 4.50±11.92 |
|  | Third-party  compensation (DUE) | -14.83±11.93 |
|  | Third-party  acceptance (AUE) | -3.33±10.45 |
|  | Third-party  acceptance (DUE) | 0.67±12.02 |
|  | Third-party  punishment (AUE) | -1.67±13.50 |
|  | Third-party  punishment (DUE) | 14.17±15.82 |

*Note*: Before AUE means before advantageous unfair experience, Before DUE means before disadvantageous unfair experience; After AUE means after advantageous unfair experience, After DUE means after disadvantageous unfair experience.

**Figure S1.** Significant channels’ activation.

**
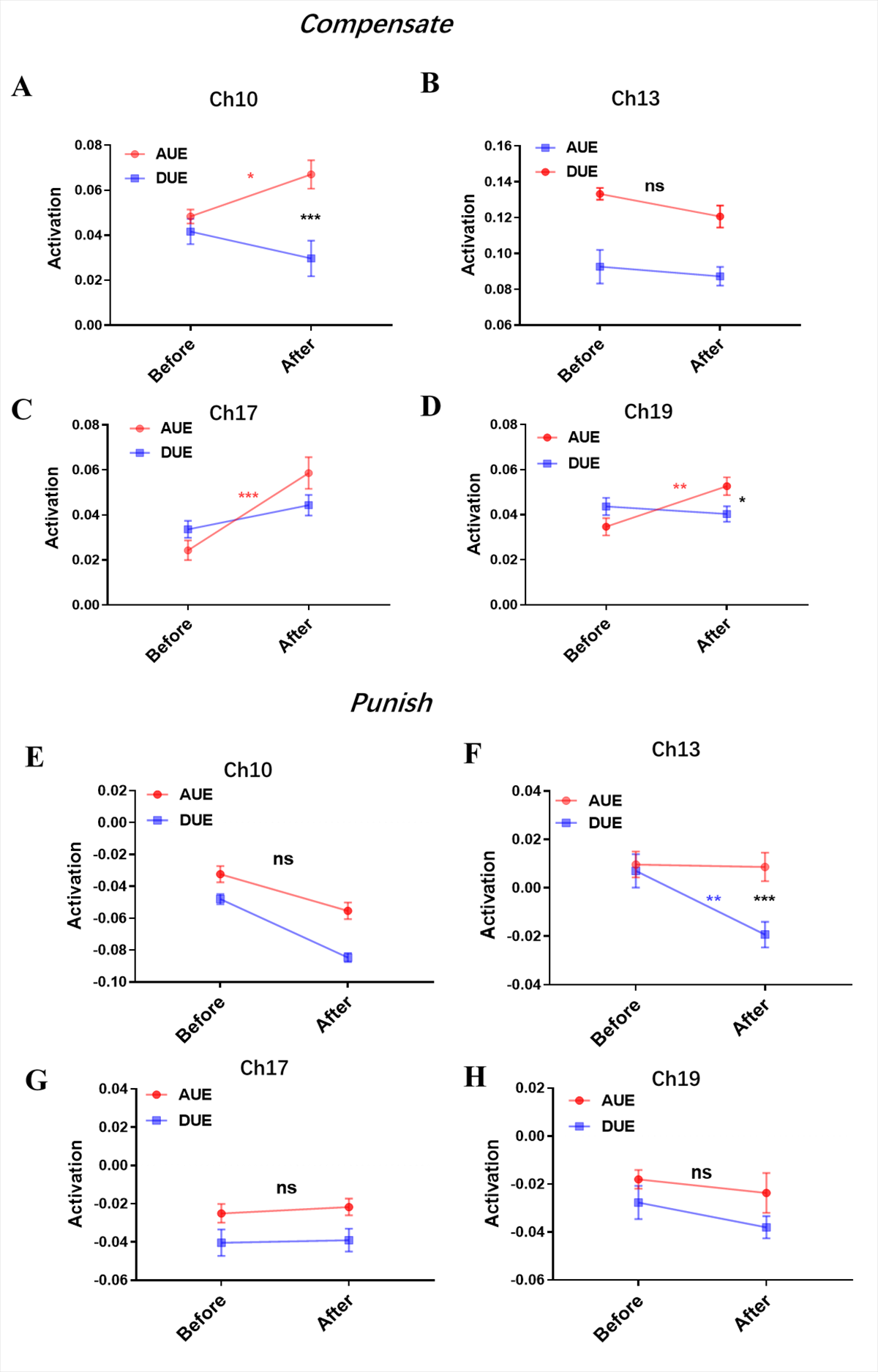
**

*Note*: ^***^*p* < 0.001, ^**^*p* < 0.01, ^*^*p* < 0.05, *ns* is non-significant. AUE means advantageous group, DUE means disadvantageous group. Before means before the unfair experience session, After before after the unfair experience session. Error bars reflect 1 SEM.

**Figure S2.** Correlation analysis examined association between the endorsement rate of *Compensate/Punish* and the brain activation before unfair experience session

**
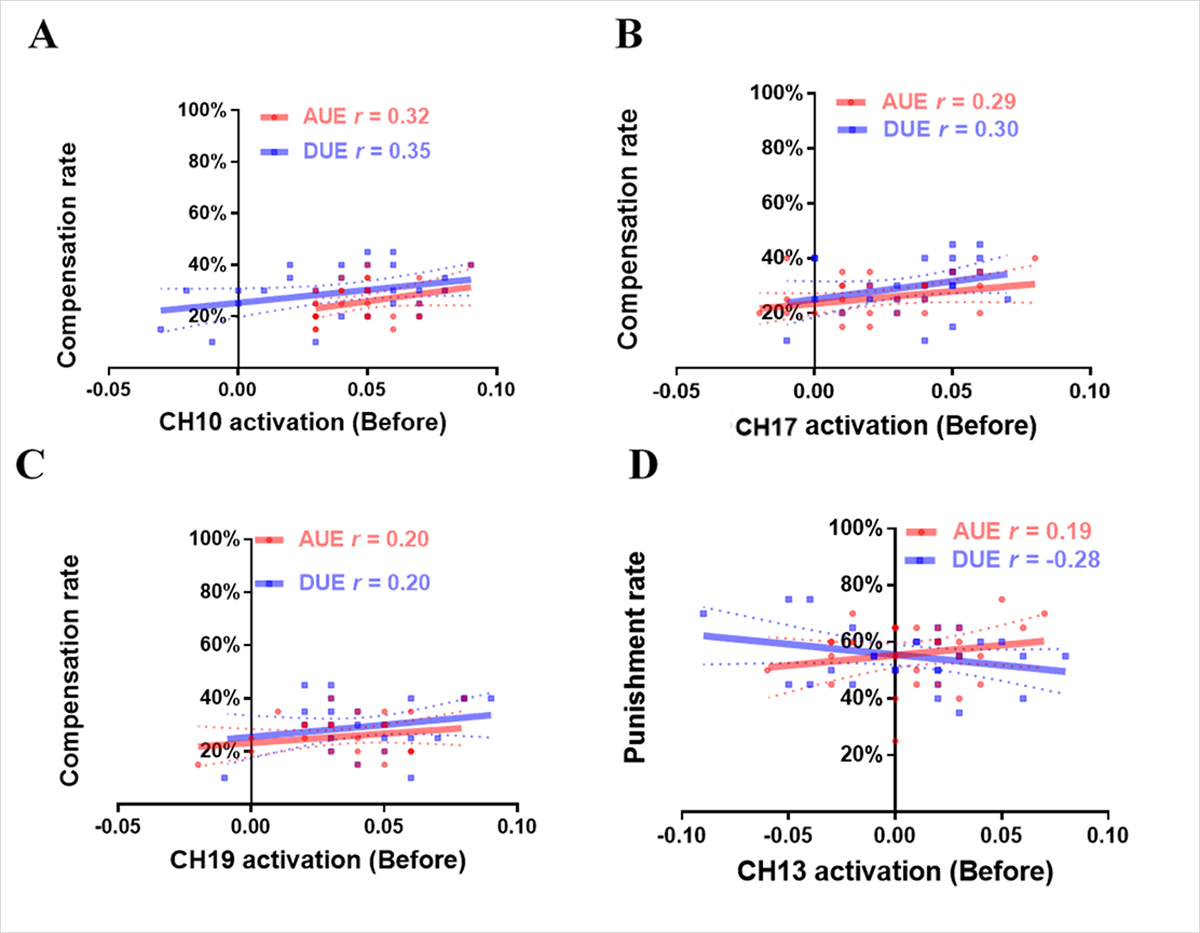
**

*Note*: The endorsement rate of third-party compensation was non-significant association with CH10 (*r*_advantageous_ _(30)_ = 0.32, *p*_advantageous_ = 0.085; *r*_disadvantageous_ _(30)_ = 0.35, *p*_disadvantageous_ = 0.069; Figure S3, A), CH17 (*r*_advantageous_ _(30)_ = 0.29, *p*_advantageous_ = 0.117; *r*_disadvantageous_ _(30)_ = 0.30, *p*_disadvantageous_ = 0.095; Figure S3, B), and CH19 (*r*_advantageous_ _(30)_ = 0.20, *p*_advantageous_ = 0.281; *r*_disadvantageous_ _(30)_ = 0.20, *p*_disadvantageous_ = 0.274; Figure S3, D) in either advantageous or disadvantageous groups under before unfair experience session. The endorsement rate of third-party punishment was non-significant correlated with the brain activation at CH13 (*r*_advantageous_ _(30)_ = 0.19, *p*_advantageous_ = 0.313; *r*_disadvantageous_ _(30)_ = -0.28, *p*_disadvantageous_ = 0.131; Figure S3, C) in either advantageous or disadvantageous groups under before unfair experience session. Before means before advantageous unfair experience or disadvantageous unfair experience.

**Figure S3**. Discrimination of third-party fairness preferences by brain activation.

**
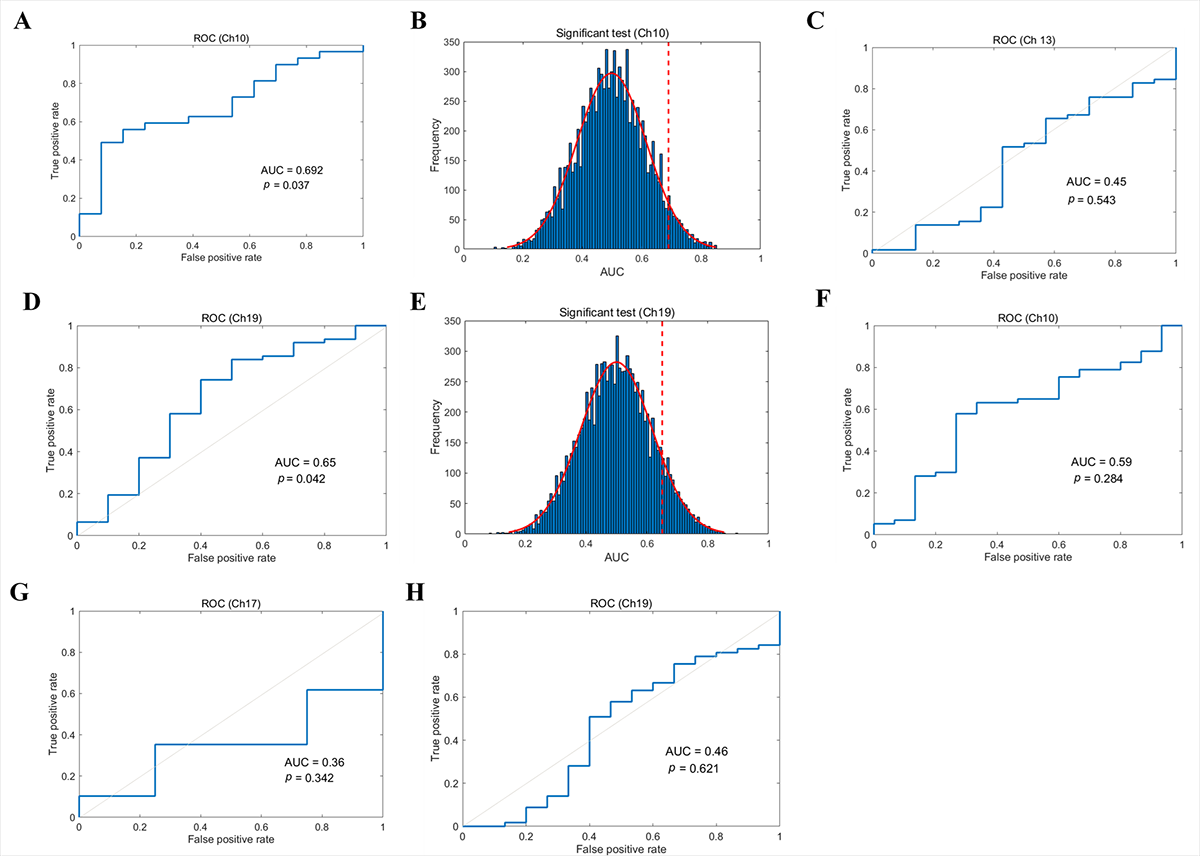
**

**Figure S4**. The mediation models that the PANAS_2 was taken as a mediator, and the sense of unfairness_2 as independent variable.

**
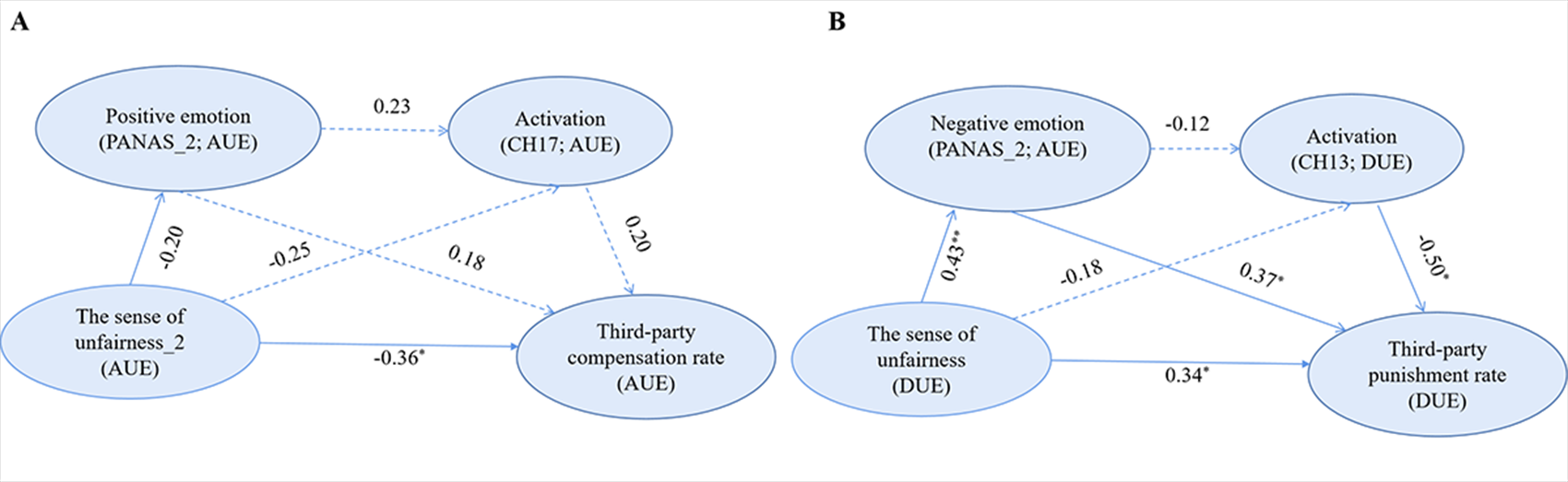
**

*Note:* (A) The results suggested a bad-fitted mediation model (CFI = 0.32, TLI = 0.45, RMSEA = 0.31) that the sense of unfairness influenced third-party compensation rate mediated by positive emotion after advantageous unfair experience. (B) The results suggested a bad-fitted mediation model (CFI = 0.36, TLI = 0.43, RMSEA = 0.30) that the sense of unfairness influenced third-party punishment rate mediated by negative emotion after disadvantageous unfair experience.
