# Supplementary figures and images for: Neural mechanisms of the mood effects on third‐party responses to injustice after unfair experiences
Source: Hum Brain Mapp. 2022 Apr 15;43(12):3646–61. doi: 10.1002/hbm.25874 (PMC9294295; doi:10.1002/hbm.25874)

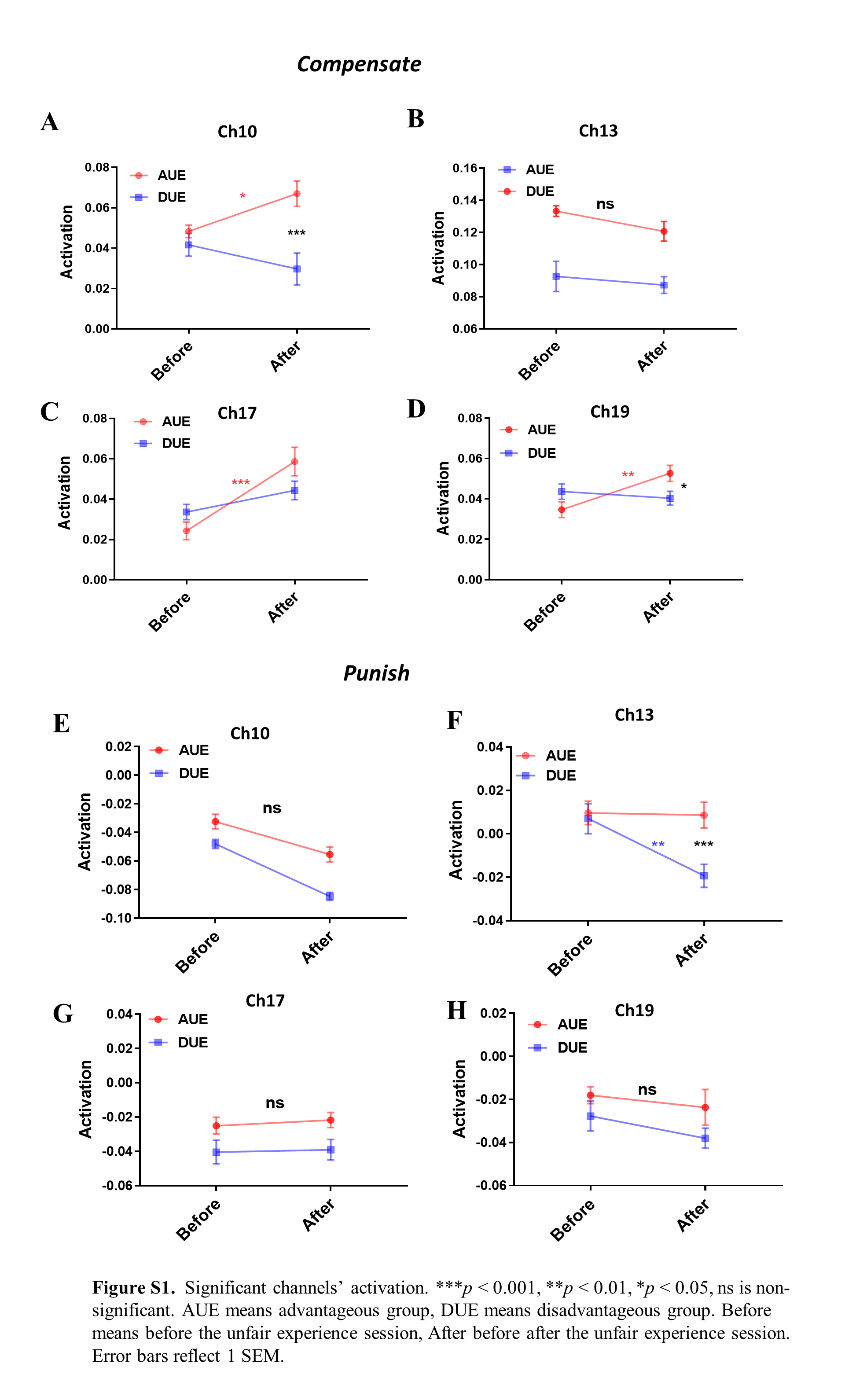

Supplement: Supplementary file 2 — Figure S1 Significant channels' activation. ***p < .001, **p < .01, *p < .05, ns is nonsignificant. AUE means advantageous group, DUE means disadvantageous group. Before means before the unfair experience session, After before after the unfair experience session. Error bars reflect 1 SEM. [file HBM-43-3646-s004.tif]

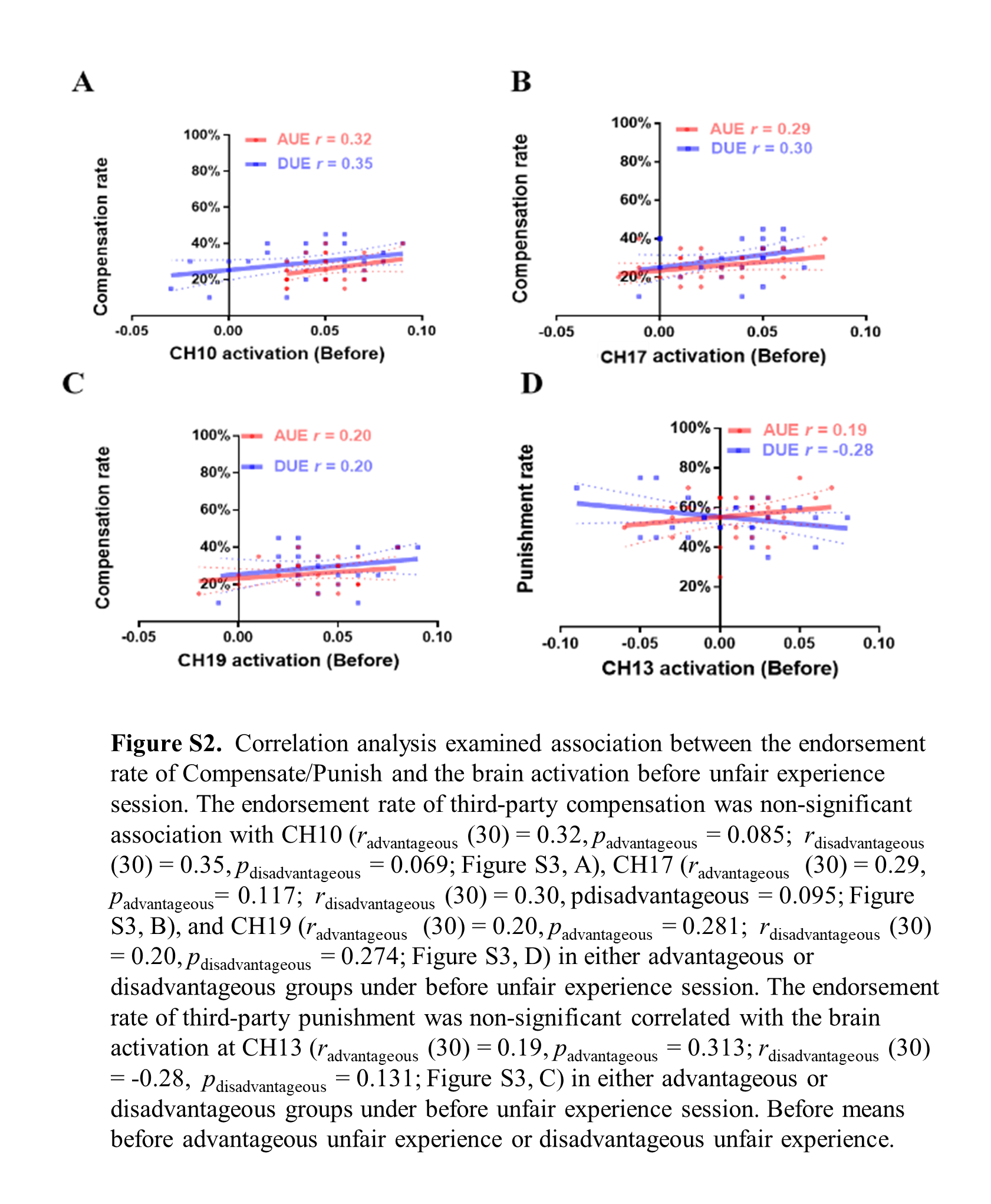

Supplement: Supplementary file 3 — Figure S2 The endorsement rate of third‐party compensation was nonsignificant association with CH10 (r advantageous (30) = 0.32, p advantageous = 0.085; r disadvantageous (30) = 0.35, p disadvantageous = 0.069; Figure S3A), CH17 (r advantageous (30) = 0.29, p advantageous = 0.117; r disadvantageous (30) = 0.30, p disadvantageous = 0.095; Figure S3B), and CH19 (r advantageous (30) = 0.20, p advantageous = 0.281; r disadvantageous (30) = 0.20, p disadvantageous = 0.274; Figure S3D) in either advantageous or disadvantageous groups under before unfair experience session. The endorsement rate of third‐party punishment was nonsignificant correlated with the brain activation at CH13 (r advantageous (30) = 0.19, p advantageous = 0.313; r disadvantageous (30) = −0.28, p disadvantageous = 0.131; Figure S3C) in either advantageous or disadvantageous groups under before unfair experience session. Before means before advantageous unfair experience or disadvantageous unfair experience. [file HBM-43-3646-s003.tif]

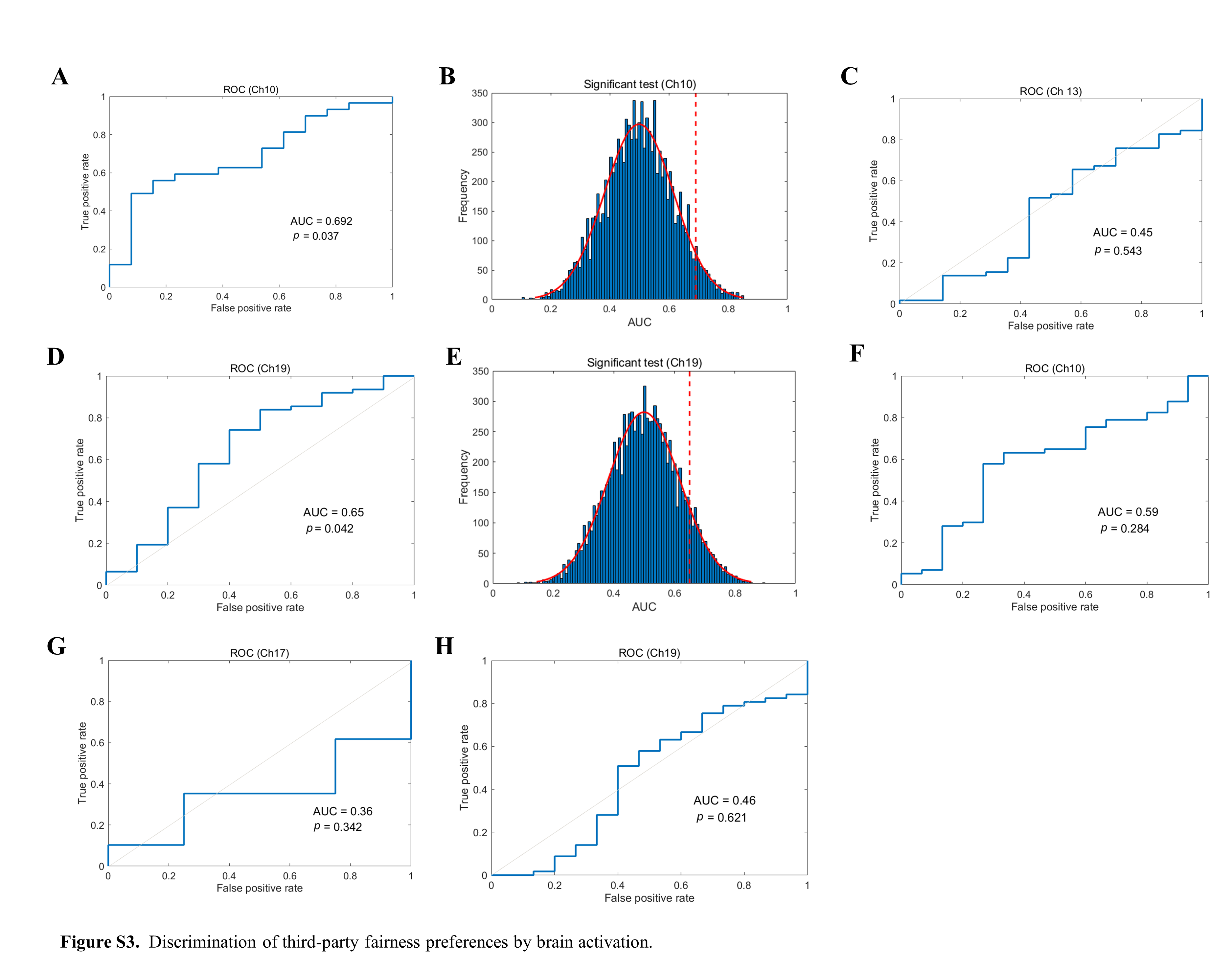

Supplement: Supplementary file 4 — Figure S3 Discrimination of third‐party fairness preferences by brain activation. [file HBM-43-3646-s001.tif]

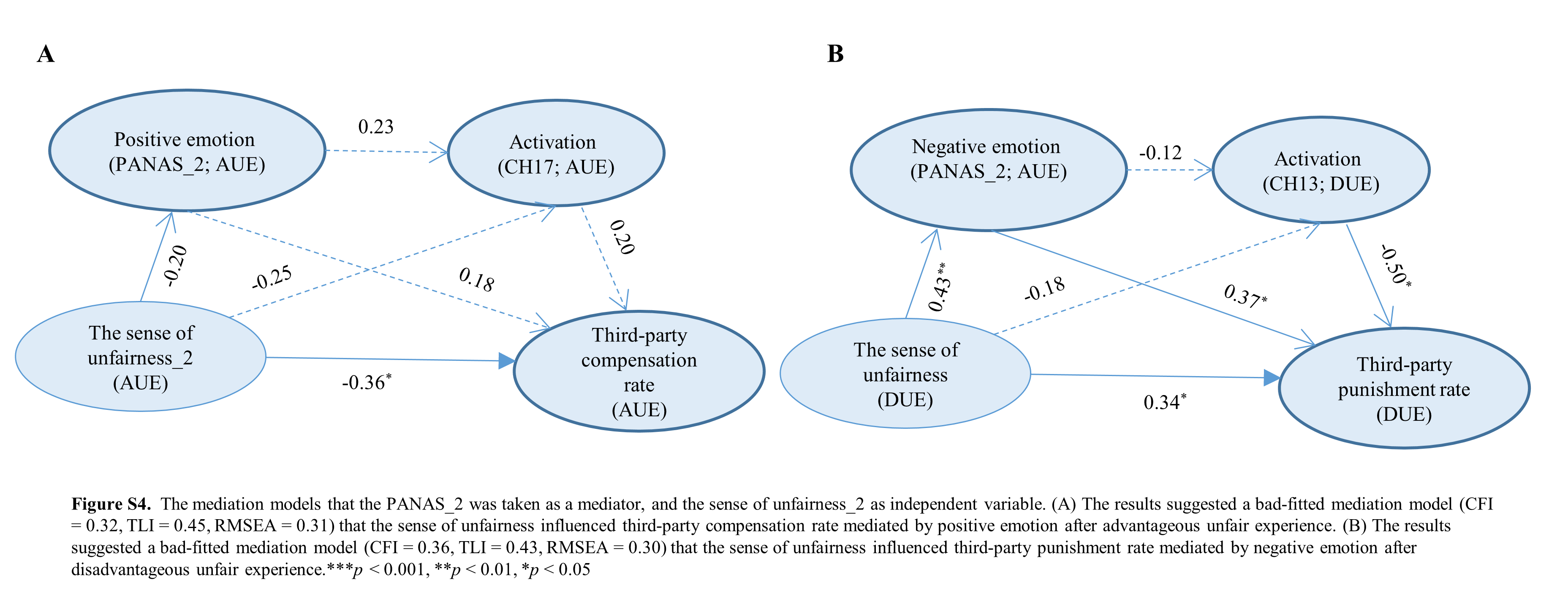

Supplement: Supplementary file 5 — Figure S4 The mediation models that the PANAS_2 was taken as a mediator, and the sense of unfairness_2 as independent variable. (A) The results suggested a bad‐fitted mediation model (CFI = 0.32, TLI = 0.45, RMSEA = 0.31) that the sense of unfairness influenced third‐party compensation rate mediated by positive emotion after advantageous unfair experience. (B) The results suggested a bad‐fitted mediation model (CFI = 0.36, TLI = 0.43, RMSEA = 0.30) that the sense of unfairness influenced third‐party punishment rate mediated by negative emotion after disadvantageous unfair experience. [file HBM-43-3646-s002.tif]
